# Peptidomimetic Lipid-Nanoparticle-Mediated Knockdown of TLR4 in CNS Protects against Cerebral Ischemia/Reperfusion Injury in Mice

**DOI:** 10.3390/nano12122072

**Published:** 2022-06-16

**Authors:** Tsogzolmaa Ganbold, Qingming Bao, Hai Xiao, Dolgorsuren Zurgaanjin, Caifeng Liu, Shuqin Han, Agula Hasi, Huricha Baigude

**Affiliations:** 1 Inner Mongolia Key Laboratory of Mongolian Medicinal Chemistry, School of Chemistry & Chemical Engineering, Inner Mongolia University, Hohhot 010020, China; tsogi001@yahoo.com (T.G.); qingming_@mail.imu.edu.cn (Q.B.); haixiao@mail.imu.edu.cn (H.X.); dolgorsuren@mail.imu.edu.cn (D.Z.); liucaifenga@yeah.net (C.L.); chem-hshq@imu.edu.cn (S.H.); 2 School of Life Sciences, Inner Mongolia University, Hohhot 010020, China

**Keywords:** lipid nanoparticles, siRNA delivery, microglia, neuroinflammation, stroke

## Abstract

Ischemic stroke activates toll-like receptor 4 (TLR4) signaling, resulting in proinflammatory polarization of microglia and secondary neuronal damage. Herein, we report a novel lipid-nanoparticle (LNP)-mediated knockdown of TLR4 in microglia and amelioration of neuroinflammation in a mouse model of transient middle cerebral artery occlusion (tMCAO). siRNA against TLR4 (siTLR4) complexed to the novel LNP (siTLR4/DoGo310), which was based on a dioleoyl-conjugated short peptidomimetic (denote DoGo310), was readily internalized by the oxygen–glucose-deprived (OGD) mouse primary microglia, knocked-down TLR4, and polarized the cell to the anti-inflammatory phenotype in vitro. Systemic administration of siTLR4/DoGo310 LNPs in the tMCAO mice model resulted in the accumulation of siRNA mainly in the Iba1 positive cells in the peri-infarct. Analysis of the peri-infarct brain tissue revealed that a single injection of siTLR4/DoGo310 LNPs led to significant knockdown of TLR4 gene expression, reversing the pattern of cytokines expression, and improving the neurological functions in tMCAO model mice. Our data demonstrate that DoGo310 LNPs could be a promising nanocarrier for CNS-targeted siRNA delivery for the treatment of CNS disorders.

## 1. Introduction

Neuroinflammation is the major cause of secondary brain injury after stroke [1]. Microglia, the resistant immune cells in central nervous systems (CNS), are the first defense line after brain injury. In response to cerebral ischemia, microglia activation appears to be a double-edged sword, which can be categorized into two opposite types: neuroprotective M2 phenotype and neurotoxic M1 phenotype [2]. M1-phenotype microglia release multiple proinflammatory factors, including TNFα, IL-1β and reactive oxygen species (ROS), via induction of a transcriptional reprogramming of genes that promote posttranscriptional activation of inflammatory signaling pathways that control the stability of nuclear factor-κB (NF-κB) and hypoxia-induced factor 1 (HIF-1) to exacerbate secondary brain injury [3], while those of M2 phenotype release anti-inflammatory factors such as IL-10, TGF-β and IL-4 to promote brain recovery after ischemic stroke [4]. In brain hypoxia during stroke, sustained neuroinflammation by overactivated M1 microglia damages neuron and cerebrovascular endothelial cells, which induces tissue destruction and worsens functional outcome [5]. Therefore, microglia are thought to be a key target for effective therapeutic intervention against ischemic stroke by regulating M1/M2 phenotypes after ischemic stroke [6].

Although small molecules [7,8] and antibody drugs [9] have been explored for anti-inflammatory therapy for CNS disorder, clinical application of short-interference RNA (siRNA) for stroke therapy has not been realized. The clinical translation of RNA interference (RNAi) technology hit a major milestone in recent years with FDA approval of three siRNA-based drugs [10], i.e., ONPATTRO (Patisiran) infusion for the treatment of peripheral nerve disease (polyneuropathy) caused by hereditary transthyretin-mediated amyloidosis (hATTR) [11,12], GILVAARI (givosiran) injection for the treatment of acute hepatic porphyria (AHP) [13,14], and OXLUMO (lumasiran) for the treatment of primary hyperoxaluria type 1 (PH1) to lower oxalate in urine [15]. However, major challenges, including development of efficient delivery systems still remain in realizing the full potential of RNAi-mediated therapeutics in clinic [16]. Lipid nanoparticles (LNP) are the only delivery system approved by FDA for siRNA drug delivery in clinic to date [17]. ONPATTRO is a formulation comprised of cationic lipid Dlin-MC3-DMA (MC3) as the key siRNA-encapsulating ingredient of the LNP structure [14]. However, the synthetic cationic head group of lipids may induce potential immune response through interaction with cell surface or cytoplasmic receptors. Although such immune response can be beneficial for antitumor therapeutics [18], it is not desirable for most other therapeutic purposes [19].

The delivery of any drugs, including siRNA, to the brain is a challenge in the treatment of CNS disorders, including ischemic stroke. The major obstruction to CNS drug delivery is the blood–brain barrier (BBB), which limits the access of drugs to the brain parenchyma. By targeting glucose transporter 1 (GLUT1), which is highly expressed in brain capillary endothelial cells, an siRNA against BACE1 was delivered to mouse brain by a galactose functionalized polymeric nanoparticle [20,21]. A poly(lactic-co-glycolic acid) (PLGA)-based nanoparticle modified with cationic lipids and detergents was able to deliver siRNA in a mouse model of traumatic brain injury (TBI) [22]. However, these nanoparticles were nonspecific for brain cell types. The brain is a heterogeneous tissue containing neurons and glial cells including astrocytes, microglia and oligodendrocytes. Each cell has unique functions in physiological and pathological conditions. Therefore, targeting a specific brain cell type that is involved in a certain neurological disorder is crucial for the development of effective therapeutics.

Previously, we designed a new class of biocompatible short peptidomimetics (denoted DoGo) for systemic delivery of siRNA against disease-related genes [23,24,25]. In this report, we formulated DoGo LNPs using four novel DoGo lipids as well as the previously designed DoGo lipids and evaluated the efficiency of CNS-targeted siRNA delivery in MCAO mouse models. Our goal is to (1) determine the distribution of DoGo LNP-mediated systemically delivered siRNA in the ischemic brain, (2) establish the brain cell-type specificity of DoGo LNPs and (3) evaluate the effect of RNAi-mediated silencing of toll-like receptor 4 (TLR4) on the stroke-induced neuroinflammation. TLRs are involved in cerebral ischemia/reperfusion (I/R) injury. A previous study revealed that in TLR4 knockout mice, neurological function was maintained, resulting from decreased brain infarct size, while in TLR2 knockout mice, higher mortality, decreased neurological function as well as increased brain infarct size were observed, demonstrating that TLR4 contributes to cerebral I/R injury [26]. Previous studies revealed that cerebral I/R injury activates TLRs, resulting in either protective or detrimental effects [27]: a sublethal ischemic injury activates TLRs, leading to an enhanced cerebral ischemic tolerance, while ischemia-induced tissue injury induces activation of TLRs and further damages tissue via the NF-κB signaling pathway [28]. Drugs targeting TLRs such as TLR3 and TLR4 have been proven to be neuroprotective against cerebral I/R injury [29].

## 2. Experimental Section

### 2.1. Chemicals and General Methods

Fmoc-Orn(Boc)-OH, Cbz-Orn(Boc)-OH, Boc-Orn(Cbz)-OH, Boc-Orn(Boc)-OH, H-Glu(Boc)-OH, *N*-(3-(dimethylamino)propyl)-*N*′-ethylcarbodiimide hydrochlorides (EDC), trifluoroacetic acid (TFA) were purchased from Aladdin (Pudong, Shanghai, China). Boc-Orn[(Boc-Orn(Boc))-OH and Boc-Orn[(Boc-Orn(Boc-Orn(Boc)))]-OH were purchased from GL Biochem (Shanghai) Ltd. (Shanghai, China). Iodomethane was purchased from Energy Chemical (Shanghai, China). Hydroxybenzotriazole (HOBt) and 1H-benzotriazol-1-yloxytripyrrolidinophosphonium Hexafluorophosphate (PyBOP) was purchased from J&K Chemical (Shanghai, China). Dimethylformamide (DMF) and dichloromethane (DCM) were distilled after drying over a 4 Å molecular sieve. ^1^H NMR and ^13^C NMR were recorded on a Bruker Avance III 500 NMR spectrometer. Chemical shifts (δ = 0 ppm) were referred to TMS with the residual proton of the deuterated solvent. MS was recorded on Finnigan LCQ Advantage MAX. DoGo1, DoGo2, DoGo3, DoGo6, DoGo220, DoGo211, DoGo311 were synthesized according to the previous reports [25] (Supplementary Materials Methods).

### 2.2. Assessment of siRNA-Binding Efficiency of DoGo Peptidomimetics

The gel electrophoresis assay was carried out to evaluate the electrostatic interactions between DoGo peptidomimetics and siRNA. Stock solution of siRNA (1 μL, 100 μM) was complexed with different amounts of DoGo peptidomimetics and incubated at r.t. for 20 min. The ratio between DoGo peptidomimetics and siRNA was calculated based on the molar number of free-amino group on DoGo (denotes N) and phosphate group on siRNA (denotes P). The N/P ratios of 0, 0.5, 1, 1.5, 2, 2.5, 3, 3.5, 4, 4.5 and 5 were tested to assess the siRNA-binding ability of DoGo220 and DoGo310 peptidomimetic. Electrophoresis was performed in 0.5× Tris borate ethylenediaminetetraacetic acid (TBE) running buffer at 100 V for 20 min. After the gel was stained using 0.5 μg/mL ethidium bromide, the gel images were taken by Gel Logic 212 PRO imaging system (Carestream, Toronto, ON, Canada).

Fluorescence-quenching assay was performed to measure the K_d_ value of DoGo220 and DoGo310. FITC-siRNA (2.5 μL, 50 μM) was diluted in 1 mL of PBS buffer (pH 7.4) in a cuvette. A total of 10 μL increments of DoGo220 or DoGo310 solution (0.2 mg/mL) was continuously added to the cuvette, recording the intensity of fluorescence signal after each increment using Fluorescence Spectrophotometer F-7000 (Hitachi, Tokyo, Japan) Fluorescence Spectrometer FLS980 (Edinburgh Instruments, EI, Livingston, UK).

### 2.3. Formulation of DoGo3 LNP

DoGo310 LNP was prepared according to the previously reported method [25]. Briefly, a solution of DoGo310, cholesterol, 1,2-distear-oyl-sn-glycero-3-phosphocholine (DSPC), and mPEG DSPE (Aladdin, Shanghai, China) in the molar ratios of 50:38.5:10:1.5 in ethanol was added to citrate buffer (50 mM, pH 4.0) while vigorously stirring. The resulting suspension contained 30% of ethanol (by volume), and the concentration of total lipids was 6.0 mg/mL. After a short equilibration at room temperature, the suspension was extruded through a membrane (pore size: 50 nm) by using an Avanti Mini-Extruder (Avanti Polar Lipids, Alabaster, AL, USA) at room temperature. A solution of siRNA in citrate buffer containing 30% ethanol was added to the DoGo310 lipoplex suspension by adjusting the weight ratio between siRNA and DoGo310 lipoplex at 0.08, followed by incubation at 35 °C for 30 min and subsequent dialysis in PBS buffer pH 7.4 for 12 h.

Size distribution of DoGo3 LNP was determined by Dynamic Light Scattering (DLS) with Zetasizer Nano-ZS90 (Malvern Instrument, Malvern, UK). The measurement was carried out at a constant temperature of 25 °C by fixing the scattering angle at 90°. The sample solution was diluted in 1× PBS (pH = 7.4) prior to analysis. Zetal potential of DoGo3 LNP dispersion system was measured by Laser Doppler Micro-electrophoresis (LDM) with Zetasizer Nano-ZS90 at 25 °C. The sample was suspended in 1× PBS (pH = 7.4) prepared with Rnase-free water prior to analysis.

### 2.4. Transmission Electron Microscopy (TEM)

Shape and surface morphological examination of siRNA/DoGo310 complex were observed by transmission electron microscopy (Field Electron and Ion Company, Hillsboro, OR, USA). After mixture of DoGo310 peptidomimetics and siRNA (N/P ratio: 5) was incubated at r.t. for 20 min, two drops of sample were placed on a copper grid (200 mesh) and then air-dried for 10 min, followed by negative staining with 2% phosphomolybdic acid solution for 2 min. The grid was allowed to air-dry for 10 min and the morphology of the resulting LNPs was examined under the TEM (Instrument: FEI Tecnai G2 F20. Voltage: 200 kV).

### 2.5. Stability and Hemolytic Assay

The serum stability of DoGo peptidomimetics complexed with siRNA was performed in 50% fetal bovine serum (FBS). The mixture of siRNA/DoGo complex and FBS was incubated at room temperature. At different time points (0 h, 0.5 h, 1 h, 2 h, 4 h, 6 h, 12 h and 24 h), a portion of the mixture was loaded on agarose gel, and electrophoresis was performed and the visible siRNA bands were quantified by UVP ChemStudio (Analytic Jena, Upland, CA, USA).

To determine the hemolytic effect, mouse red blood cells (2%) were incubated with DoGo peptidomimetics or DoGo peptidomimetics/siRNA complex in PBS buffer at different concentrations of DoGo peptidomimetics. PEI was used as comparison group, and PBS as well as 1% Triton X 100 were used as negative and positive control, respectively. After incubation for 2 h at 37 °C, the mixture was centrifuged at 2200 rpm for 10 min to obtain the supernatant. The transmittance at 450 nm was determined by FilterMax F5 Multi-Mode Microplate Reader (Molecular Devices, San Jose, CA, USA). The hemolysis was calculated with the following formula:

Hemolysis (%) = (A_sample_ − A_PBS_)/(A_Triton_ − A_PBS_) × 100% (1)

### 2.6. Isolation and Culturing of Mouse Primary Microglia

Primary microglia were isolated from P3 neonatal mouse pups according to the previous report [30]. Briefly, the pups were sacrificed by decapitating and the heads were immediately dropped into 70% ethanol. After rinsing the heads in saline solution, the whole brain was removed from the heads and was placed into a chilled Leibovitz’s L-15 conditioned media (Leibowitz L-15 + 0.1% BSA + 1% Penicillin/Streptomycin) on ice. The brain tissue was smashed by pipetting and the suspension was passed through a 70 μm cell strainer (Bector Dickinson, Franklin Lakes, NJ, USA). After the cells were centrifuged at 160× *g* for 10 min, the supernatant was discarded, and the cell pellet was resuspended in culture media (DMEM + 10% FBS + 1% Penicillin/Streptomycin). After transferring to a cell-culture plate (Costar, Corning Inc., Somerville, MA, USA), the cells were incubated at 37 °C with 95% air/5% CO_2_. The cells were first incubated for 5 days, and on the fifth day, the media were replaced with the fresh culture media. The media were replaced in every 3 days, and after 3 weeks the cells were utilized for further studies.

### 2.7. Cytotoxicity Measurement

BV2 cells (ATCC, Manassas, VA, USA) were maintained at 37 °C (with 5% CO_2_) in DMEM supplemented (10% fetal bovine serum) with 100 mg mL^−1^ streptomycin and 100 U mL^−1^ penicillin. BV2 cells or primary microglia were grown in a 96-well plate and the cells were treated with various concentrations of DoGo peptidomimetics on the next day. After 24 h, the cellular toxicity was assessed using MTS assay.

### 2.8. Cellular Uptake and Plasmid-Delivery Activity

BV2 cells or microglia were plated into 12-well plates (CORNING, Corning, NY, USA) at a density of 1.0 × 10^5^ cells (BV2) or 1.0 × 10^6^ cells (primary microglia) 24 h before the cellular uptake assay. DoGo peptidomimetics complexed with fluorescent labeled siRNA (FITC-siRNA, custom synthesized by Takara Biotechnology Co., Ltd., Dalian, China) were added to the cells, and the cells were then incubated for 6 h. After the incubation, the cells were washed with 1× PBS (pH 7.4) till complete removal of extracellular FITC-siRNA. The cells were fixed with 4% paraformaldehyde at r.t. for 20 min and then the fixed cells were probed with primary antibodies for microglia marker Iba1 (5 mg/mL, Abcam, Cambridge, UK) and visualized with secondary antibodies, i.e., goat antirabbit Alexa Fluor ^®^ 594 conjugate which was used along with the nuclear marker DAPI (Thermo Scientific, Waltham, MA, USA) according to manufacturer’s instructions. After the cells were washed three times with PBS (1×, pH 7.4) at room temperature, the coverslips were detached and mounted on a glass slide by mounting media and the cellular uptake of the complex by primary microglia was captured by confocal microscopy (Olympus, Fluoview FV 1000). For quantification of cellular uptake, the cells were trypsinized and washed with PBS (1×, pH 7.4) to remove extracellular FITC-siRNA. After treating the cells with complexes of FITC-siRNA/DoGo peptidomimetics, the cells were applied for flow cytometer assay. As a comparison group, commercial transfection reagent Lipofectamine 2000 (L2K) were used.

To evaluate delivery efficiency of DoGo peptidomimetics, 293T cells were seeded in 24-well culture plates (Corning-Coaster, Tokyo, Japan). When the cell growth achieved 70–80% confluence, the cells were treated with the green fluorescence reporting plasmid DNA (pcDNA3-eGFP, 1 µg per well) complexed with DoGo peptidomimetics. Before transfection, the mixture (DoGo peptidomimetics/plasmid) was incubated at r.t. in 100 mL of serum-free Opti-MEM medium for 20 min and was added to the cells. After 72 h incubation, the results were captured by fluorescence microscopy. To compare the transfection efficiency of DoGo220 and DoGo310, luciferase assay was performed using pGL3-SV40 plasmid (Promega, Madison, WI, USA), following the manufacturer’s protocol.

### 2.9. siRNA-Delivery Efficiency on Primary Microglia

For in vitro delivery of siRNA, primary microglia were treated with siRNA against TLR4 (siTLR4) and the resulting RNAi activity was measured by RT-qPCR and Western blot analysis. siGenome-nontargeting siRNA (siCtrl) (Dharmacon, Lafayette, CO, USA) was used as a control. TLR4 siRNA was custom-synthesized by Takara Bio-technology Co., Ltd. (Dalian, Liaoning, China). The primary microglia were seeded into a 12-well plate 24 h before siRNA transfection. siTLR4 (100 nM) or siCtrl (100 nM) and DoGo LNP (20 μg/mL) were mixed in Opti-MEM (100 μL) and was incubated at r.t. for 20 min. After adding 400 μL of fresh complete medium into the mixed solution, the mixture was added to the cells. The transfected cells were incubated for 4 h and were subsequently treated with OGD (Oxygen–glucose deprivation) insult, according to the previous report [31]. To perform OGD insult, cells were washed three times in DMEM without glucose and FBS, and then the culture medium was replaced with low-glucose DMEM. Cells were cultured for 2 h at 37 °C in an airtight incubation chamber with a continuous flux of gas (5% CO_2_ and 95% N_2_). OGD was then relieved by removing the culture from the incubator and maintaining the cells in high-glucose medium and reoxygenated for 24 h under normal conditions (37 °C, 5% CO_2_, 95% air at high humidity). The sequences of the TLR4 siRNAs are:

Antisense strand: 5′-UUAUAGUCAAAUAUGGGCCtt-3′;

Sense strand: 5′-GGCCCAUAUUUGACUAUAAtt-3′.

For RT-qPCR analysis, total RNA was extracted from the cells using TRIZOL reagent (Invitrogen, Carlsbad, CA, USA) and the expression of endogenous TLR4 mRNA was measured using iScript^TM^ Reverse Transcription Supermix for RT-qPCR and iTaq^TM^ Universal SYBR Green Supermix (Bio-Rad, Hercules, CA, USA). The sequences of the primers used for RT-qPCR were list in Table S1. For Western blotting, total protein was extracted with a protein-extraction kit (CWBio, Beijing, China) containing antiproteases and antiphosphatases (CWBio, Beijing, China). Protein concentration was determined using the BCA protein assay kit (Thermo Scientific, Waltham, MA, USA). A total of 10 μg of total protein was run on an SDS-PAGE gel and then transferred to a nitrocellulose membrane (Amersham, Orsay, France). Membranes were blocked for 1 h with TBS/0.1% Tween 20 (TBST) with 5% skim milk. Membranes were incubated for 1 h at r.t. with primary antibodies (anti-GAPDH or anti-TLR4 antibody) purchased from Cell Signaling Technology (Danvers, MA, USA), diluted according to the provider’s recommendations in TBST with 2% skim milk. After washing with 2% skim milk 3 times (each 10 min), the membranes were incubated with secondary antibodies (Abcam, Cambridge, UK) at 1/5000 dilution in TBST with 2% milk for 1 h, and the signal was revealed with SuperSignal^®^ West Pico (Thermo Scientific, Waltham, MA, USA) using UVP ChemStudio (Analytic Jena, Upland, CA, USA).

### 2.10. In Vitro Microglia Polarization

After knockdown of TLR4 in OGD-modeled microglia, the expression of canonical markers for M1 and M2 polarization was measured by qPCR and ELISA. Commercially available ELISA kits (Thermo Scientific, Waltham, MA, USA) were used to determine the levels of IL-1β, TNF-α, IL-10 and IL-4 in the culture media according to manufacturer’s instructions.

M1 and M2 marker expression in microglia was also examined using immunocytochemistry (ICC) according to previously published methods [32,33]. Microglia were treated with siTLR4/DoGo310 for 4 h, followed by OGD treatment. After incubating the cells in normal cell-culturing condition for 24 h, the cells were fixed in 4% PFA. Fixed cells were probed with primary antibodies for the M2 phenotype (CD206, Cell Signaling Technology, Danvers, MA, USA) or the M1 phenotype (iNOS, Cell Signaling Technology, Danvers, MA, USA), and then for visualization, the secondary antibodies, antirabbit Alexa-594 (Cell Signaling Technology, Danvers, MA, USA) or goat antirabbit IgG-FITC (Abcam, Cambridge, MA, USA) were used along with the nuclear marker DAPI according to manufacturer’s instructions. Images were captured by sequential scanning of the immunostained tissues with an Olympus Fluorescence confocal microscope (Olympus, Fluoview FV 1000).

### 2.11. In Vitro Rescue of Degenerating Neurons

After microglia were transfected with siTLR4/DoGo310 or siCtrl/DoGo310 LNP followed by OGD modeling (detailed in Section 2.9 siRNA-transfection efficiency on primary microglia), the cell-culture media (i.e., conditioned media) were harvested and added into precultured neuron cells (N2a) and incubated for 24 h. Then, the cell viability was measured using calcein AM/PI double-staining kit (Yeasen, Shanghai, China) and CellTiter 96^®^ Aqueous one-solution cell-proliferation assay (Promega, Madison, WI, USA), respectively, following the manufacturer’s instructions.

### 2.12. CNS Targeted Delivery of Fluorescently Labeled siRNA

Balb/c mice (male, 10 weeks old) were purchased from Inner Mongolia University Experimental Animal Center. All animal experiments were approved by the Animal Care and Use Committee of the Inner Mongolia University (Approval number: 21875124). The transient middle cerebral artery occlusion (tMCAO) was performed as described previously, except for filament insertion [34,35]. In brief, the mice were anesthetized with an intraperitoneal injection of 350 mg/kg chloral hydrate. After a midline skin incision, the left external carotid artery was exposed, and its branches were ligated. A nylon monofilament (diameter = 0.16 mm) coated with silicon (diameter = 0.23 mm) was introduced into the internal carotid artery through the common carotid artery and advanced until faint resistance was felt. After 90 min of occlusion, blood flow was restored by withdrawing the nylon thread to allow reperfusion. Sham-operated control mice received the same procedure except for filament insertion. Three days after the modeling, FITC-siRNA/DoGo310 complex (dose of siRNA: 1 mg/kg body weight; dose of DoGo LNP: 15 mg/kg body weight) was administered via intravenous (i.v.) injection. Twenty-four hours after the i.v. injection, the mice were sacrificed, and the brain was collected for detection of distribution of FITC-labeled siRNA in the brain. Briefly, the brain slides (3 mm) were fixed and then probed with primary antibodies microglia marker Iba1 (5 mg/mL) and visualized by secondary antibodies (goat antirabbit Alexa Fluor 594 conjugate) along with the nuclear marker DAPI in a similar way described in Section 2.8. Images were captured by an Olympus Fluorescence confocal microscope.

### 2.13. In Vivo Delivery of TLR4 siRNA in tMCAO Mouse Model

Experimental groups were designed as follows:

I. Group 1 (sham group), 6 healthy mice that underwent sham surgery.
II. Group 2 (tMCAO group), 6 tMCAO model mice that received 90 min of ischemia and reperfusion for 72 h.
III. Group 3 (siTLR4/DoGo310 LNP treatment group), 6 mice that initially received 90 min of ischemia and reperfusion for 3 days and then were injected with siTLR4/DoGo310 LNP by i.v. injection.
IV. Group 4 (siCrtl/DoGo310 LNP treatment group), 6 mice that initially received 90 min of ischemia and reperfusion for 3 days and then were injected with siCtrl/DoGo310 complex by i.v. injection.

After dividing the groups, Group 3 and Group 4 were intravenously injected with siTLR4/DoGo310 or siCtrl/DoGo310 LNP, respectively. The dosages of DoGo310 and siRNA were 15 mg/kg and 1 mg/kg body weight, respectively. Seventy-two hours after administration of siTLR4/DoGo310 LNP, the mice were sacrificed. For siRNA-delivery efficiency, total RNA and total protein were isolated from the peri-infarct tissue, and qPCR and Western blotting were performed to detect the expression of TLR4 at mRNA and protein levels, respectively. For microglia polarization, total RNA and total protein extracted from peri-infarct were used. For immunohistochemistry analysis, the brain slide was probed with primary antibodies for microglia marker, Iba1 or neuron marker, NeuN (Abcam, Cambridge, UK) and visualized by secondary antibodies, goat antirabbit Alexa Fluor 594 conjugate and goat antirabbit IgG-FITC were used along with the nuclear-marker DAPI. Images were acquired by sequential scanning of the immunostained tissues with an Olympus Fluorescence confocal microscope. In tissue slide, NeuN-positive cells were quantified, counting green fluorescence cells in the same 6 areas of the slides for each group.

### 2.14. Assessment of Infarct Size and Evaluation of Neurological Deficits

For assessment of infarction, hematoxylin and eosin (H&E) staining was conducted in a similar method that has been published previously [35]. Triphenyl tetrazolium chloride (TTC) staining was performed according to the previous report [36]. Neurological functional scores were evaluated using the modified Bederson scale [37]. Scores range from 0 for no deficits, 2 for flexed forepaw, 4 for inability to resist lateral push, 6 for circling, 8 for agitated circling and 10 for unresponsiveness to stimulation/stupor.

### 2.15. Statistical Analyses

Statistical significance was determined using one-way analysis of variance with a Dunnett’s multiple comparison test. *p*-values of <0.05 were considered statistically significant. The analysis was performed using GraphPad Prism 7.0.

## 3. Results and Discussion

### 3.1. Synthesis and Screening of DoGo Peptidomimetics

**Scheme 1:**
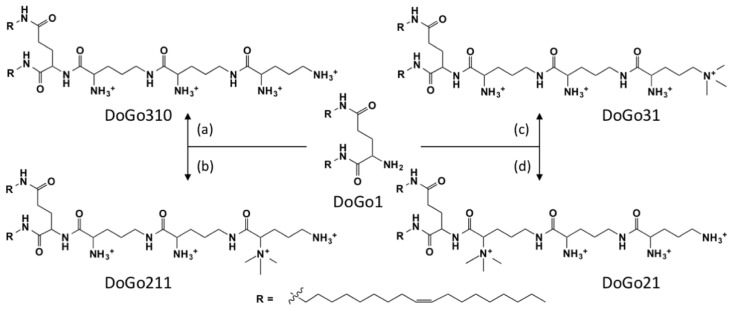
Synthesis of novel DoGo peptidomimetics. (**a**). (*i*) Boc-Orn[(Boc-Orn(Boc-Orn(Boc)))]-OH, PyBOP, DIPEA; (*ii*) HCl/1,4-dioxane. (**b**). (*i*) Cbz-Orn(Boc)-OH, PyBOP, HOBt; (*ii*) TFA/DCM; 3 repeats of (*i)–(ii*). (*iii*) CH_3_I, K_2_CO_3_; (**c**). (*i*) Cbz-Orn(Boc)-OH, PyBOP, HOBt; (*ii*) TFA/DCM; 2 repeats of (*i)–(ii*). (*iii*) Boc-Orn(Cbz)-OH, PyBOP, HOBt; (*iv*) TFA/DCM; (*v*) CH_3_I, K_2_CO_3_. (**d**). (*i*) Fmoc-Orn(Boc)-OH, EDCI, TEA; (*ii*) DEA/DMF; (*iii*) CH_3_I, K_2_CO_3_; (*iv*) TFA/DMF; (*v*) Boc-Orn[(Boc-Orn(Boc))]-OH, EDCI, DIPEA; (*vi*) HCl/1,4-dioxane.

To create an efficient and biocompatible carrier for CNS-targeted delivery of therapeutic siRNAs, we designed a class of cationic lipid molecules, in which short peptidomimetics with unique peptide linkages function as a cationic head group (Scheme 1). The novel lipids (denote DoGo peptidomimetics) were synthesized using standard organic-synthesis procedures (Scheme S1, Figures S1 and S2). A preliminary assessment of size and surface charge of particles originated from DoGo lipids after encapsulating siRNA revealed that the diameter of the complexes ranged from 50 nm to 150 nm, with zeta potential ranging from 22 mV to 35 mV (Figure S3A,B). To test the cytotoxicity of novel lipids (DoGo310, DoGo31, DoGo211, DoGo21) as well as the previously designed DoGo lipids on glial cells, we treated BV2 cells (mouse microglia cell line) with DoGo lipids, respectively, and measured the cell viability by MTS assay. At the same concentration, DoGo310 and DoGo220 showed the highest cell viability compared to all other lipids (Figure S3C). Meanwhile, we evaluated the in vitro microglia-targeted siRNA-transfection ability of each individual DoGo lipids. To achieve this, we treated BV2 cells with the DoGo lipids complexed with FITC-labeled siRNA (FITC-siRNA) against GAPDH, respectively. After 24 h, we quantified the fluorescence-positive cells using flow-cytometry analysis, and subsequently measured the mRNA level of GAPDH by RT-qPCR. The results showed that although all DoGo lipids exhibited more than 40% of FITC-positive cells, some failed to induce RNAi against endogenous gene expression (Figure S3D). For example, cells treated with DoGo21, DoGo31 and DoGo211 complexed to FITC-siRNA showed good flow-cytometric fluorescence intensity but did not exhibit a gene-silencing effect. Because flow cytometry counts all cells that have fluorescence signals regardless of the FITC-siRNA attached on the cell surface or internalized into the cytoplasm, we conclude that DoGo lipids differ in their microglial cell-transfecting efficacy due to their differing penetrating efficiency and possibly the endosomal escape capability. Because DoGo220 and DoGo310, isomers of same molecular formula design, both bearing three ornithine residues in the head group with either linear or branched linkage, showed the highest siRNA-transfection efficiency and lowest cytotoxicity, we chose these two lipids for further comparison for the suitability for in vitro and in vivo siRNA delivery to glial cells.

**Figure 1:**
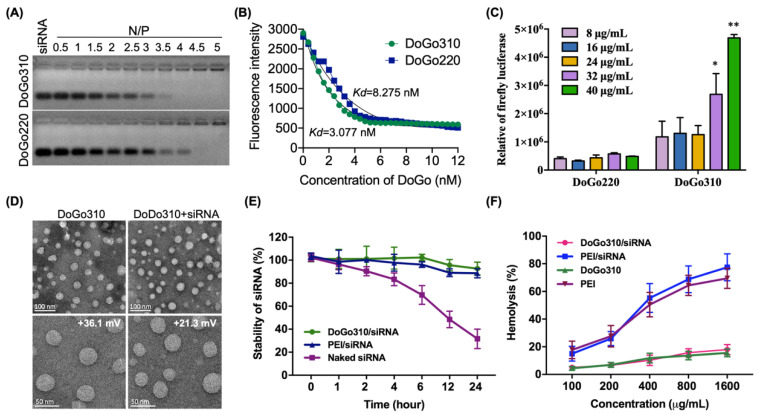
Physicochemical properties of DoGo310. (**A**) Assessment of siRNA-binding affinity by gel electrophoresis assay; (**B**) apparent dissociation constant (*K_d_*) of DoGo220 and DoGo310 lipids with siRNA measured by fluorescence quenching assay; (**C**) comparison of plasmid-delivery efficiency of DoGo220 and DoGo310 lipids. All data are presented as mean ± SD from triplicate experiments; * *p* < 0.05, ** *p* < 0.001 compared to untreated cells; (**D**) Morphology of DoGo310 lipid after formulation. TEM image shows size of the DoGo310 LNP after siRNA loading; (**E**) siRNA protection efficiency of DoGo310 LNP. Naked siRNA and siRNA loaded to PEI are shown as control; (**F**) hemolysis assay of DoGo310 LNP alone or loaded with siRNA.

In our previous work, we used DoGo220 lipid, which bears a head group with branched amino groups, as a helper lipid for cholesterol-based Chorn3 lipids for the study of tumor-targeted siRNA delivery [38]. Interestingly, gel-electrophoresis assay demonstrated that the newly synthesized DoGo310 lipid, which has a linear head group, encapsulated siRNA at lower ratio of lipid/siRNA (indicated by nitrogen/phosphate ratio, denotes N/P) compared to DoGo220 (Figure 1A). We further measured the apparent dissociation constant (i.e., *K_d_*) of both lipids, and found that the *K_d_* value of DoGo310 (3.07 nM) in binding siRNA is much lower than that of DoGo220 (8.27 nM). The reduced *K*_d_ value indicates that the DoGo310 has a significantly higher siRNA-binding affinity than DoGo220 (Figure 1B). It is possible that the formation of branched structure in head group of a cationic lipid could potentially augment spatial hindrance that may negatively affect binding affinity with nucleic acids, whereas linear structure minimizes the spatial hindrance and facilitate the electrostatic interaction. A previous report also revealed that linear structure in the hydrophobic tail of LNPs has more capacity to delivery siRNA to leukocytes compared to the branched pattern [14].

To compare the plasmid-transfection efficiency of DoGo310 and DoGo211 lipids, we treated 293T cells with the lipids complexed to pcDNA3-eGFP plasmid, respectively. The result showed that the transfection with pcDNA3-eGFP/DoGo310 complex resulted in significantly higher GFP-expressing cell population compared to pcDNA-eGFP/DoGo220 (Figure S4). A parallel evaluation of transfection efficiency using a plasmid-expressing firefly luciferase (pGL3-SV40) also showed that DoGo310 lipid has significantly higher transfection efficiency compared to DoGo211 (Figure 1C). Based on these observations, we chose DoGo310 lipid for further formulation and evaluation of microglia-targeted siRNA delivery in vitro and in vivo.

### 3.2. Formulation and In Vitro Evaluation of DoGo310 LNP

Next, we formulated DoGo310 lipid into LNPs, using cholesterol, 1,2-distear-oyl-sn-glycero-3-phosphocholine (DSPC), and mPEG DSPE (Aladdin, Shanghai, China) in the molar ratios of 50:38.5:10:1.5, according to the previous report [39].The particle size of formulated DoGo310 ranged from 30 nm to 50 nm, with zeta potential of +21.3 mV after siRNA loading (Figure 1D). Meanwhile, siRNA loaded to DoGo310 LNP efficiently resisted hydrolysis by serum nuclease in a similar efficiency to polyethylenimine (PEI) (Figure 1E) but with significantly lower hemolysis effect (Figure 1F). These data suggest that DoGo310 LNP has a favorable particle size as an siRNA carrier, with remarkable siRNA encapsulation and protection efficiency, as well as negligible hemolysis effects.

**Figure 2:**
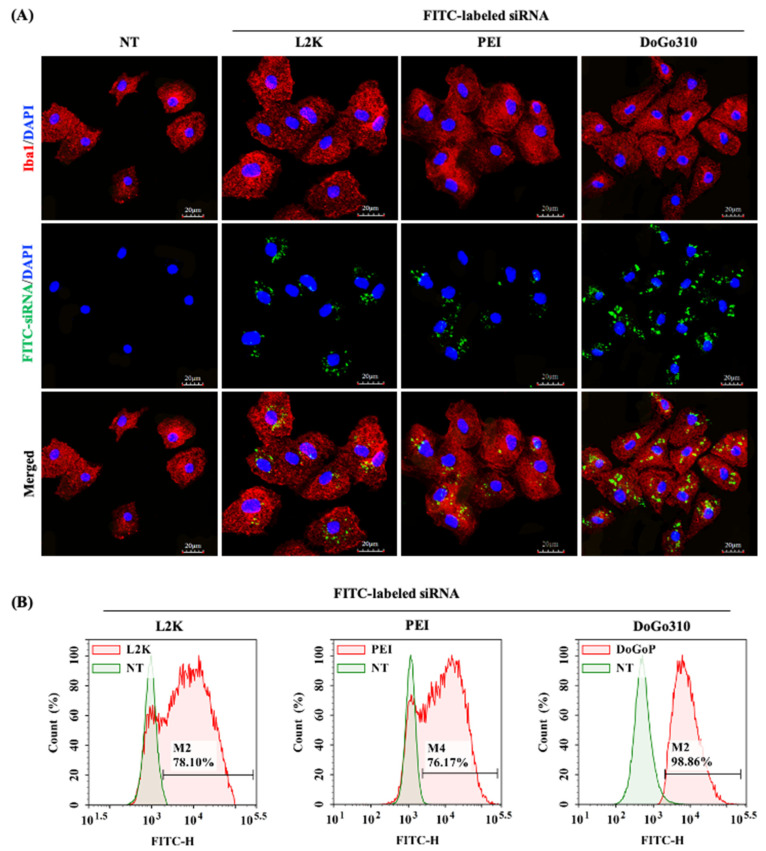
DoGo310 LNPs deliver fluorescently labeled siRNA more efficiently compared to Lipofectamine 2000 (L2K) and PEI. (**A**) Confocal images of Iba-1 positive mouse primary microglia treated with FITC-siRNA/L2K, FITC-siRNA/PEI, or FITC-siRNA/DoGo310, respectively. Red, anti-Iba-1 antibody; blue, DAPI; green, FITC; NT, nontreated. Scale bar = 20 mm. (**B**) Quantification of FITC positive cells by flow cytometry.

Next, we chose mouse primary microglia cells for the in vitro glial cell model for evaluation of DoGo310 LNP-mediated siRNA delivery. To achieve this, we first isolated mouse primary microglia. Immunocytochemistry analysis using anti-Iba1 antibody revealed the purity of the isolated cells (Figure 2A). The fluorescence image of primary microglia cells treated with FITC-siRNA complexed to DoGo310 LNP showed strong signals of fluorescence in the cytoplasm (Figure 2A). Flow-cytometry analysis revealed that compared to Lipofectamine 2000 (L2K) and PEI, DoGo310 LNP has a much higher uptake efficiency of FITC-siRNA by microglia (Figure 2B).

**Figure 3:**
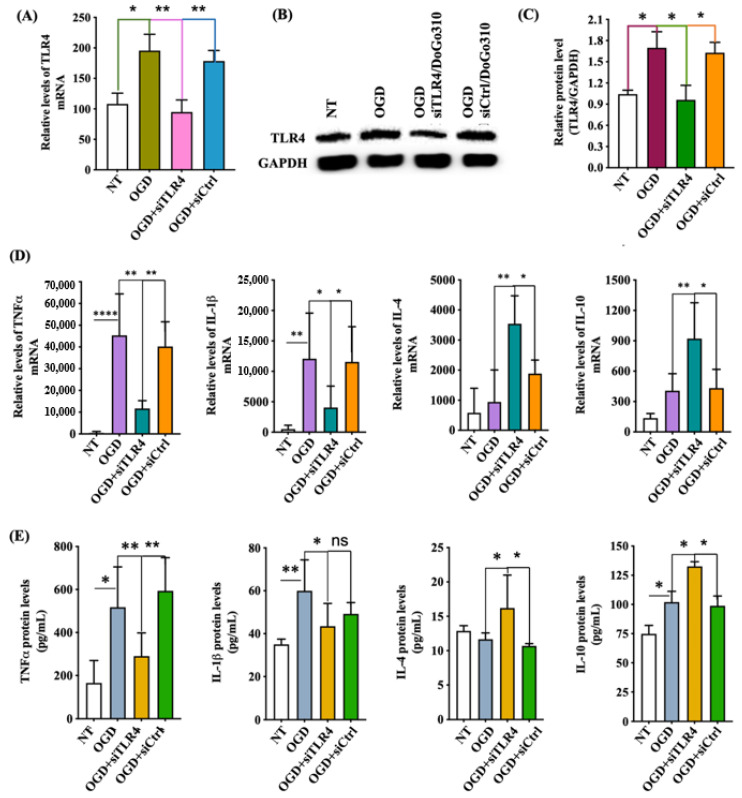
Mouse primary microglia polarizes to anti-inflammatory M2 phenotype through inhibiting TLR4 by siTLR4/DoGo310 complex. (**A**–**C**) Analysis of target gene expression by RT-qPCR and Western blot after transfection of siTLR4/DoGo310 complex; (**D**,**E**) silencing of TLR4 in OGD-treated microglia results in decrease in key proinflammatory cytokines, while resulting increase in anti-inflammatory cytokines, as analyzed by RT-qPCR and ELISA. NT, nontreated. NS, Not Significant. * *p* < 0.05, ** *p* < 0.001, **** *p* < 0.00001 compared to untreated cells.

To verify the gene-silencing capability of siRNA/DoGo310 LNPs in oxygen–glucose-derivation (OGD)-modeled microglia, which mimics in vitro classically activated microglia that contribute to neuronal apoptosis via releasing proinflammatory cytokines during ischemic stroke [3], we used siRNA against toll-like receptor 4 (siTLR4). TLR4 is one of the first-line molecules for initiating an innate immune response after brain injury, including ischemic stroke. TLR4-dependent microglia activation leads to neuroinflammation and neuronal loss in ischemic stroke [40]. The TLR4/nuclear factor-κB (NF-κB) pathways are critical to the molecular modulation of cellular phenotype. Stimulation of TLR4 can further activate the NF-κB pathway and play an important role in the activation of M1 phenotype microglia [41]. It has been established that the inhibition of TLR4 reduced hypoxia-induced upregulation of proinflammatory cytokines, including TNF-α, IL-1β, iNOS and ROS in microglia cells[40]. OGD treatment significantly upregulated TLR4 expression in primary microglia cells, mimicking proinflammatory polarization (M1 activation) in vitro. Treatment with siTLR4/DoGo310 LNPs inhibited the expression level of TLR4 in OGD-treated microglia at both mRNA (Figure 3A) and protein levels (Figure 3B,C), while the expression level of TLR4 was not affected by the complex of DoGo310 and double-stranded random-sequenced RNA (siCtrl). Furthermore, measurements of pro- and anti-inflammatory cytokines released by OGD-treated microglia before and after siRNA transfection by RT-qPCR and ELISA revealed that treatment of OGD-modeled microglia with siTLR4/DoGo310 complex downregulated the expression of proinflammatory cytokines such as TNFα and IL-1β, and upregulated the expression of anti-inflammatory cytokines including IL-4 and IL-10 at both the mRNA and protein level (Figure 3D,E). These data suggested that DoGo310 LNPs can efficiently transfect siRNA into microglia in vitro, and the knockdown of TLR4 may promote the alternative polarization of OGD treated microglia, resulting in the alteration of the cytokine release pattern of microglia.

### 3.3. Analysis of Microglia Polarization via DoGo310 LNP-Mediated siRNA Delivery In Vitro

**Figure 4:**
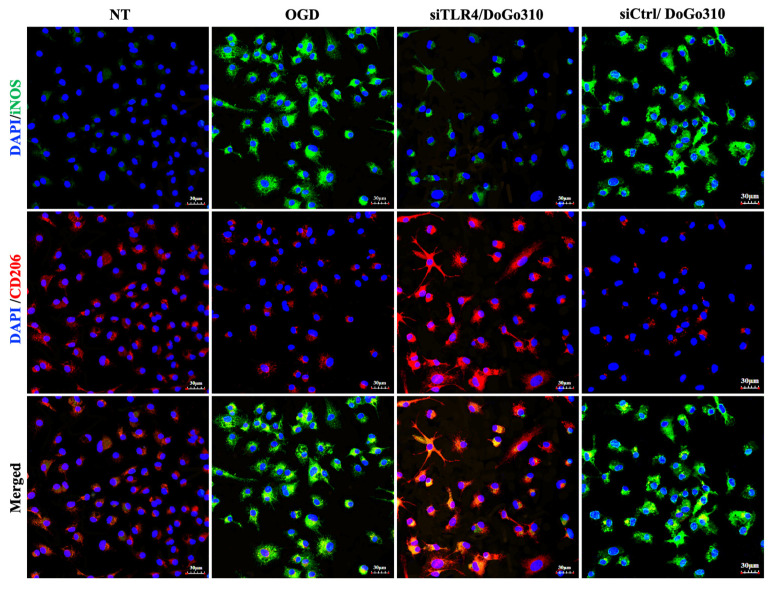
DoGo310 LNP-mediated delivery of siTLR4 reverse microglia polarization in vitro. Immunocytochemistry analysis showing the downregulated expression of M1 polarization marker iNOS (green), and upregulated expression of M2 polarization marker CD206 (red) in siTLR4/DoGo310 LNP-treated cells but not in siCtrl/DoGo310 LNP-treated cells. NT, nontreated.

**Figure 5:**
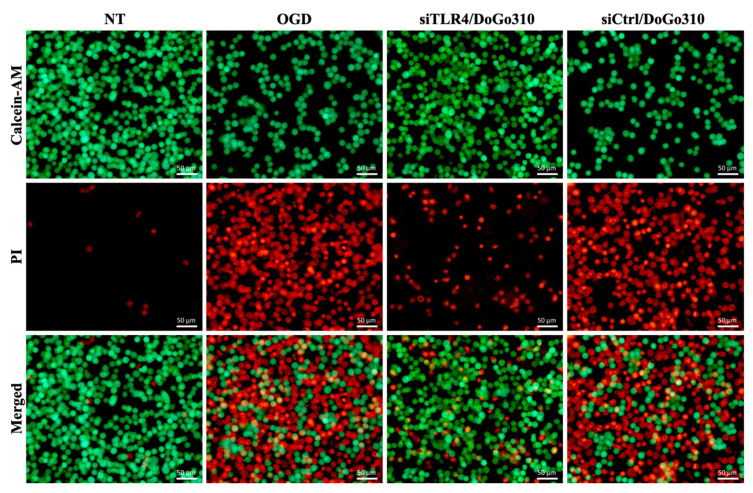
DoGo310 LNP-mediated silencing of TLR4 in OGD-treated microglia reduces neurotoxicity. OGD-treated microglia were treated with either siTLR4/DoGo310 LNPs, or siCtrl/DoGo310 LNPs. Then, the culture media was harvested from each group and was added to N2a cells. Red represents dead cells stained by PI; green represents live cells stained with Calcein-AM. NT, nontreated. Scale bar = 50 mm.

To test the possible phenotype changes induced by siTLR4/DoGo310 LNP treatment, we examined microglia under confocal microscope after immunostaining with typical markers for M1 or M2 polarization. As shown in Figure 4, the expression of CD206 (M2 marker), which had a basic level in NT and OGD group, was significantly upregulated in the siTLR4/DoGo310-treated group, while iNOS (M1 marker), which was dramatically upregulated by OGD treatment, was significantly downregulated only in the siTLR4/DoGo310 group. Furthermore, we investigated the in vitro neuroprotective effect of siTLR4/DoGo310-treated microglia. The conditioned medium harvested from the culture of siTLR4/DoGo310-treated microglia showed apparently fewer apoptotic N2a cells relative to the siCtrl/DoGo310-treated group (Figure 5). These results suggested that DoGo310-mediated knockdown of TLR4 led to the downregulation of proinflammatory cytokines and upregulation of anti-inflammatory cytokines, resulting in the phenotypic switch of microglia, which exerted reduced toxicity on neurons under hypoxic condition via inhibition of TLR4 expression.

### 3.4. CNS-Targeted siRNA Delivery by DoGo310 LNPs

**Figure 6:**
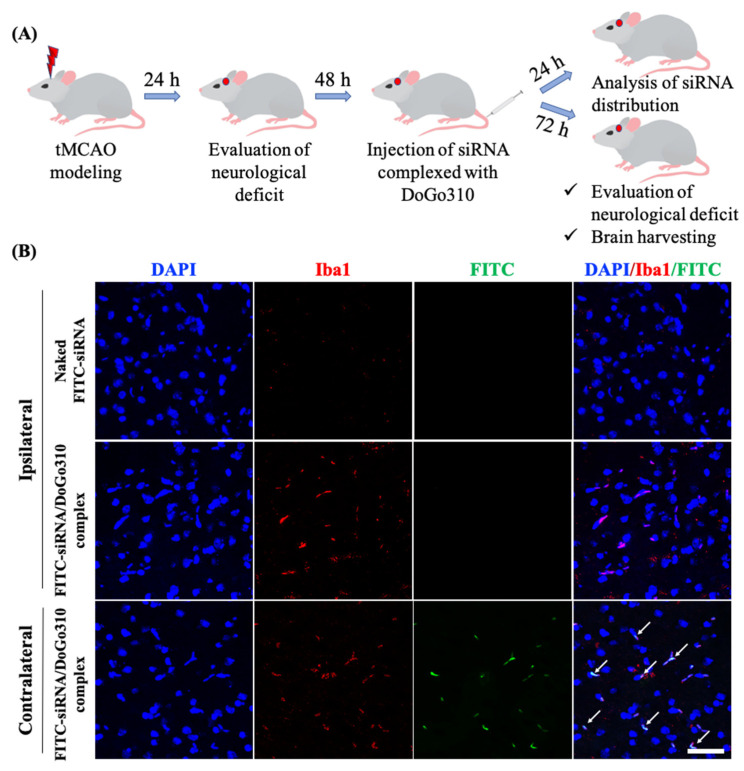
In vivo siRNA delivery by DoGo310 LNPs in tMCAO mouse. (**A**) Schematic illustration for siRNA administration and analytical process. (**B**) Immunohistochemistry staining showing the distribution of siRNA in peri-infarct region of contralateral as well as ipsilateral brain of tMCAO model mouse after i.v. injection of an FITC-label siRNA complexed to DoGo310 LNPs (dose of siRNA: 1.0 mg/kg body weight). Naked FITC-siRNA was injected as a negative control. Green, FITC-siRNA; red, Iba1. Scale bar = 50 µm.

With the acquisition of these compelling in vitro results, we moved forward to in vivo experiments using the transient middle cerebral artery occlusion (tMCAO) mouse model. To examine brain distribution of siRNA delivered by systemic administration of DoGo310 in tMCAO mouse, we administered FITC-siRNA/DoGo310 via tail-vein injection to the mouse at 24 h after tMCAO modeling (Figure 6A). A strong fluorescence signal of FITC-labeled siRNA was detected in the peri-infarct area, mostly colocalizing with Iba1-positive microglia cells (Figure 6B), suggesting that the systemically injected siRNA/DoG310 complex penetrated into the brain via leaky BBB in the ischemic brain region, and was taken up by microglia. The ipsilateral cortex did not exhibit fluorescence signal after injection with FITC-siRNA/DoG310 complex, suggesting that only the peri-infarct region in contralateral cortex received systemically delivered siRNA.

**Figure 7:**
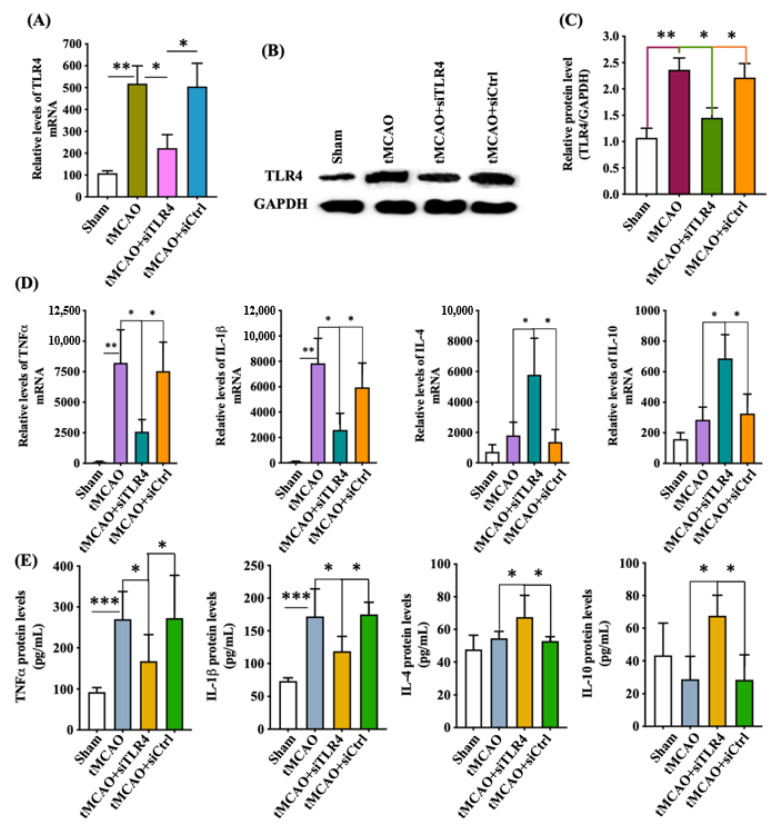
Target gene knockdown efficiency and differentiated expression of cytokines induced by DoGo310 LNP-mediated siRNA delivery in tMCAO mouse. (**A**–**C**) Analysis of target gene expression by RT-qPCR and Western blot after transfection siTLR4/DoGo310 complex; (**D**,**E**) silencing of TLR4 in OGD-treated microglia results in decrease in key proinflammatory cytokines, while resulting increase in anti-inflammatory cytokines, as analyzed by RT-qPCR and ELISA. * *p* < 0.05, ** *p* < 0.001, *** *p* < 0.0005 compared to untreated cells.

Next, we injected the tMCAO mouse with siTLR4/DoGo310 complex. Microglia activated into an anti-inflammatory state a few hours after ischemic insult, and then polarized to proinflammatory M1 state from about 72 h to days or months [42]. Because our goal was to investigate TLR4 knockdown-mediated microglia polarization, we administered siTLR4 48 h after stroke modeling. Then, 72 h after i.v. injection of siRNA/DoGo310 NPs, the whole brain was harvested for analysis of target gene expression. TLR4, which was strongly upregulated after tMCAO modeling, was significantly downregulated at both the mRNA (Figure 7A) and protein level (Figure 7B,C) in peri-infarct tissue only in the siTLR4/DoGo310 administered group, but not in the siCtrl/DoGo310 control group. Subsequent analysis of peri-infarct brain tissue showed decreased levels of proinflammatory cytokines and increased levels of anti-inflammatory cytokines at both mRNA and protein level, whereas dramatically increased level of proinflammatory cytokines and no obvious changes of anti-inflammatory cytokines were detected in mice injected with siCtrl/DoGo310 (Figure 7D,E).

### 3.5. The Neuroprotective Effect after DoGo310 LNP-Mediated Silencing of TLR4

**Figure 8:**
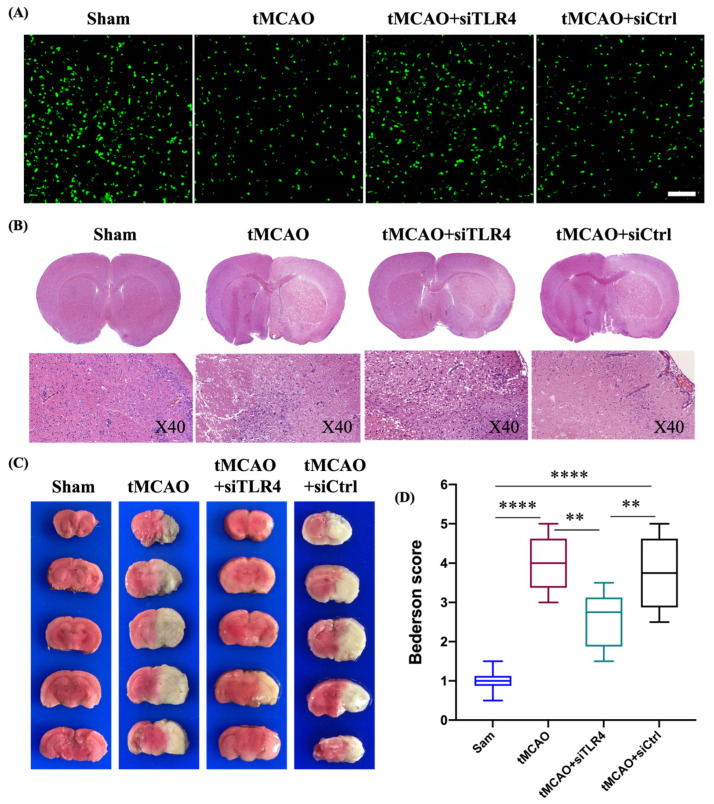
The inhibition of TLR4 by complex of siTLR4/DoGo310 attenuates cerebral I/R injury in mice at 3 d after injection. (**A**) Representative images of NeuN-positive cells in the infarct region. Scale bar = 100 mm. (**B**) H&E staining in tMCAO mouse brain with higher-magnification images (lower panel, magnification: 40×). (**C**) TTC staining of brain sections from each group of mice. (**D**) Neurological scores 72 h after administration of siRNA/DoGo310 complexes. *n* = 6 mice. ** *p* < 0.001, **** *p* < 0.00001.

To assess the possible in vivo neuroprotection induced by the silencing of TLR4 after administration of siTLR4/DoGo310 to tMCAO mice, we stained NeuN (neuronal markers)-positive cells in mouse brain slices. A decreased number of NeuN^+^ cells were detected in the infarct lesion of the nontreated tMCAO model mouse brain, while siTLR4/DoGo310 treatment mice showed significantly more NeuN^+^-positive cells in the infarct lesion (S5 and 8A). Moreover, histopathological analysis of the peri-infarct area of brain tissue examined by hematoxylin and eosin (H&E)-staining suggested that ingestion of siTLR4/DoGo310 results in reduced nuclear pyknosis and edema, and an improvement in cell density and cell morphology, where the nontreated tMCAO group or siCtrl/DoGo310 group results in ischemic injury-induced vacuolization and nuclear pyknosis, neuronal necrosis, and edema (Figure 8B). Moreover, the infarct size of ischemic brain was evaluated using 2,3,5-triphenyltetrazolium chloride (TTC) staining (Figure 8C). The knockdown of TLR4 expression resulted in the reduced size of cerebral lesion relative to other treatment groups, demonstrating the alleviation of stroke-induced tissue damage and the neuroprotective effect of the RNAi-mediated inhibition of TLR4 at the cellular and tissue levels. In addition, the inhibition of TLR4 contributed to the neurological outcome with improved neurological function (Figure 8D). Our previously study suggested that the inhibition of NF-κB in tMCAO mice regulates the expression of inflammatory mediators in ischemic stroke by modulating the microglia activation to M2 phenotype [43]. Inhibiting TLR4 in tMCAO mice may regulate the expression of inflammatory cytokines via the NF-kB pathway and result in a neuroprotective effect.

We successfully designed and synthesized novel lipid-functionalized peptidomimetics based on natural amino acids with unique peptide linkages for effective gene silencing in CNS, which is one of the most challenging issues in drug delivery and RNAi therapeutics. The new DoGo310 LNPs showed efficient silencing effect of TLR4 both in vitro and in vivo. Despite the numbers of amino groups on the head group, the linear structure of DoGo310 peptidomimetics is more efficient in siRNA delivery compared to the branched structure. Knockdown of TLR4 signaling resulted in the switch of microglial polarization, which is different from traditional anti-inflammatory therapy targeting a single factor such as IL-1 receptor [44], because RNAi against TLR4 not only inhibited the release of a group of proinflammatory cytokines, including iNOS, but also prompted the production of several anti-inflammatory cytokines and neurotrophins. Taken together, these results suggested that the novel DoGo310 LNPs could open new avenues for delivering therapeutic RNAi to CNS and may also be introduced in biomedical applications.

## 4. Conclusions

In conclusion, DoGo310 LNPs have superior binding affinity to siRNA compared to DoGo220. Such high affinity to siRNA not only resulted in excellent in vitro delivery efficiency but also led to the accumulation of siRNA specifically in microglia in the peri-infarct of tMCAO mice. Silencing of TLR4 in activated microglia by systemic administration of siTLR4 complexed to DoGo310 LNPs in tMCAO mice promoted the expression of anti-inflammatory factors, while inhibiting the proinflammatory cytokines, and contributed to the neuroprotection and recovery of neurological functions.

## Data Availability

The data that support the findings of this study are available in the Supplementary Materials of this article.

## Supplementary Materials

The following supporting information can be downloaded at: https://www.mdpi.com/article/10.3390/nano12122072/s1, Scheme S1: synthetic routes; Figure S1: results of synthesis methods; Figure S2: Chemical structure of the designed DoGo peptidomimetics; Figure S3. Initial screening of DoGo lipids for cytotoxicity and transfection efficiency; Figure S4. Evaluation of plasmid transfection efficiency of the peptidomimetics; Figure S5: siTLR4/DoGo310 LNP mediated knockdown of TLR4 rescued NeuN+ cells (neurons) in peri-infarct region of tMCAO model mouse; Table S1: The sequences of the primers used for RT-qPCR analysis.
