# Supplementary material for: Peptidomimetic Lipid-Nanoparticle-Mediated Knockdown of TLR4 in CNS Protects against Cerebral Ischemia/Reperfusion Injury in Mice
Source: Nanomaterials (Basel). 2022 Jun 16;12(12):2072. doi: 10.3390/nano12122072 (PMC9228890; doi:10.3390/nano12122072)
Supplement: Supplementary file 1 [file nanomaterials-12-02072-s001.zip › nanomaterials-1760074-supplementary.pdf]

Supplementary Materials

Peptidomimetic Lipid-Nanoparticle-Mediated Knockdown of TLR4 in CNS Protects against Cerebral Ischemia/Reperfusion Injury in Mice

Tsogzolmaa Ganbold [1,2], Qingming Bao [1], Hai Xiao [1], Dolgorsuren Zurgaanjin [1], Caifeng Liu [1], Shuqin Han [1], Agula Hasi [2,*] and Huricha Baigude [1,*]

1 Inner Mongolia Key Laboratory of Mongolian Medicinal Chemistry, School of Chemistry & Chemical Engineering, Inner Mongolia University, Hohhot 010020, China; tsogi001@yahoo.com (T.G.); qingming_@mail.imu.edu.cn (Q.B.); haixiao@mail.imu.edu.cn (H.X.); dolgorsuren@mail.imu.edu.cn (D.Z.); liucaifenga@yeah.net (C.L.); chem-hshq@imu.edu.cn (S.H.)
2 School of Life Sciences, Inner Mongolia University, Hohhot 010020, China
* Correspondence: hasind@sina.com (A.H.); hbaigude@imu.edu.cn (H.B.)

1. Experimental Methods

1.1. Synthesis of DoGo21

Synthesis of Intermediate 1:

(a) DoGo1 (200 mg, 0.31 mmol), Fmoc-Orn(Boc)-OH (211 mg, 0.46 mmol), and triethylamine (TEA) (64 μL, 0.46 mmol) were dissolved in dry dichloromethane (DCM) (5 mL) in an eggplant flask. When the reaction system reaches 0 °C on ice bath, 1-ethyl-(3-dimethylaminopropyl) carbodiimide hydrochloride (EDCI) (89.14 mg, 0.46 mmol) was added. After removing the ice bath, the reaction was stirred at room temperature (r.t.) for 24 h under the protection of N_2. Afterwards, the reaction mixture was successively washed with 5% citric acid, distilled water and saturated brine. The organic phase was collected and dried over anhydrous Na_2SO_4 and passed through silica gel column chromatography (eluent: dichloromethane/methanol = 10/1 (v/v)). (b) Removal of Fmoc group: the collected product was dissolved in a mixture of diethylamine (DEA) and dimethylformamide (DMF) (2/3, volume ratio, v/v) and stirred vigorously at r.t. for 4 h. After the solvents were removed, the obtained syrup was dissolved in DCM and was successively washed with distilled water, 5% citric acid and saturated brine, and then dried over anhydrous Na_2SO_4, followed by purification by silica gel column chromatography (eluent: dichloromethane/methanol = 8/1 (*v*/*v*)) to give 101.2 mg intermediate 1. Overall yield: 41%. The synthetic route and characterization are shown in Scheme S1.1. Figure S1.1 and Figure S1.2, respectively.

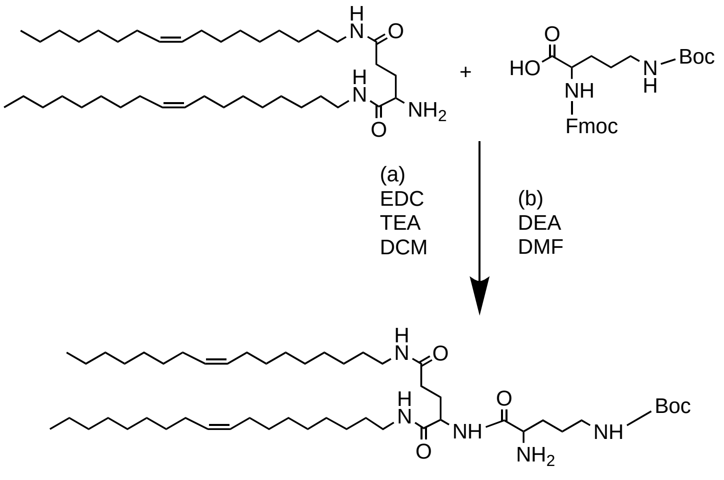

Scheme S1.1 Synthesis of intermediate 1 of DoGo21.

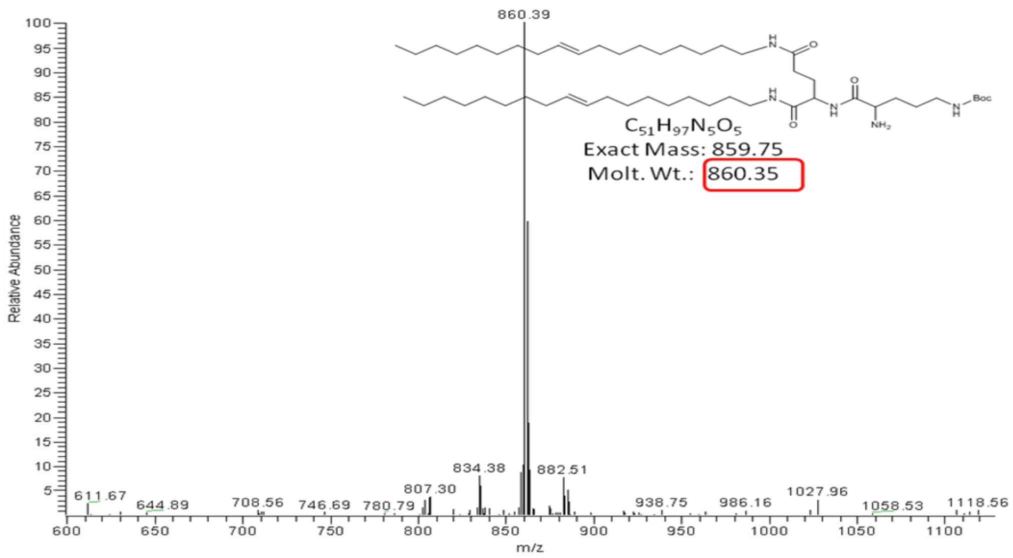

Figure S1.1 MS Spectrum of Intermediate 1 of DoGo21.

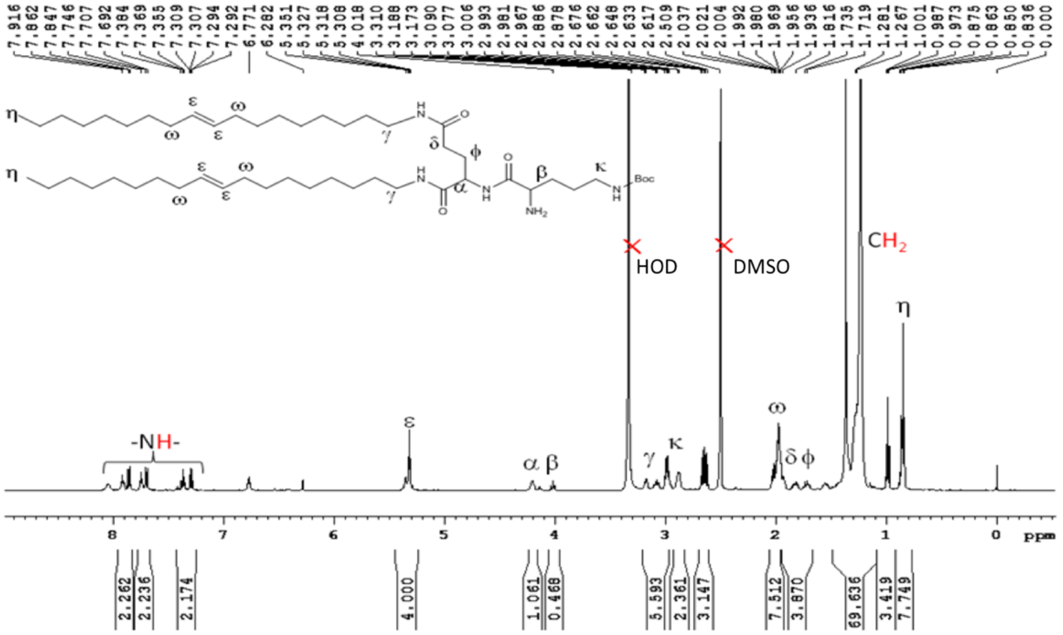

Figure S1.2 1HNMR Spectrum of Intermediate 1 of DoGo21. (Solvent: DMSO-d6).

Synthesis of Intermediate 2 of DoGo21:

(a) Intermediate 1 (199.6 mg, 0.116 mmol) was dissolved in methyl iodide (CH_3I) (4 mL). Then K_2CO_3 was added and the suspension was stirred at 40°C for 24 h under nitrogen protection. After the reaction, iodomethane was removed by distillation under reduced pressure with a rotary evaporator, and the resulting syrup was purified by silica gel column chromatography (eluent: dichloromethane/methanol = 8/1 (*v*/*v*)). (b) Deprotection: the crude product was then dissolved in a mixture of trifluoroacetic acid (TFA) and DMF (1/1, *v*/*v*) and was stirred vigorously at r.t. for 30 min, followed by concentration under reduced pressure to give 73.1 mg intermediate 2. Yield: 40%. The synthetic route and characterizations are shown in Scheme S1.2 and Figure S1.3 and Figure S1.4, respectively.

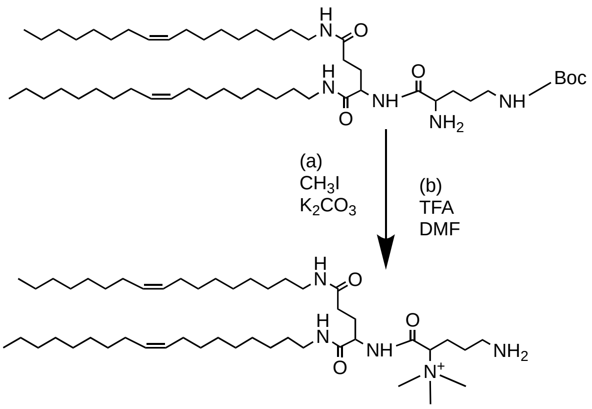

Scheme S1.2 Synthesis of intermediate 2 of DoGo21.

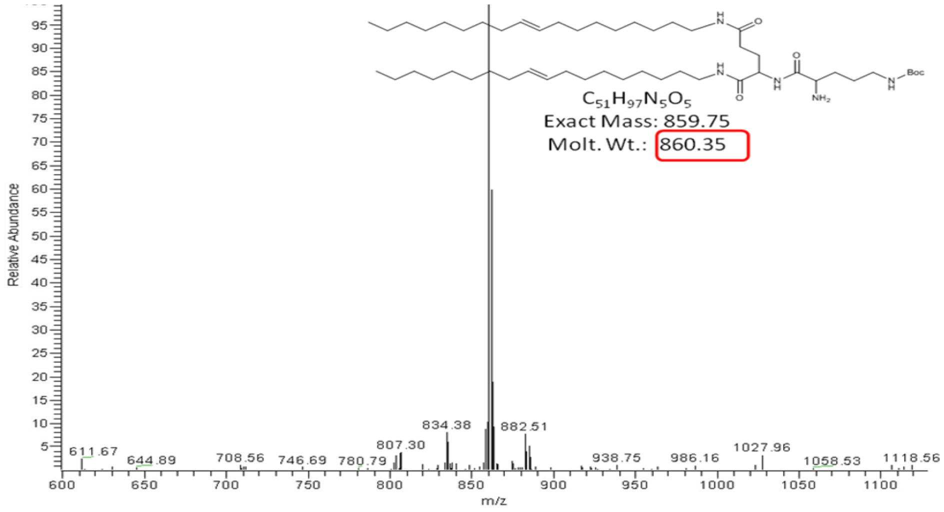

Figure S1.3 MS spectrum of Intermediate 2 of DoGo21.

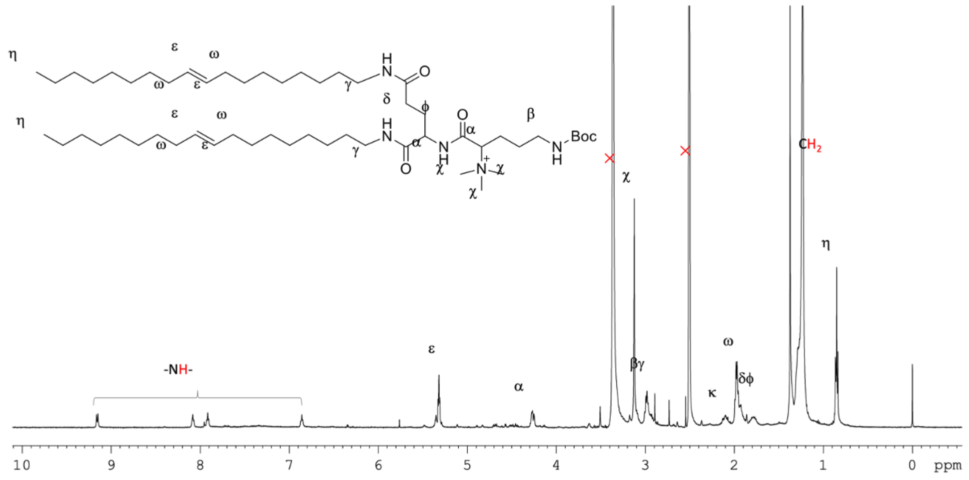

Figure S1.4 30 ^{1}HNMR spectrum of Intermediate 2 of DoGo21 (Solvent: DMSO-d$_6$).

Synthesis of DoGo21:

(a) Intermediate 2 (73.1 mg, 0.08 mmol), Boc-Orn(Boc-Orn(Boc))-OH (52 mg, 0.1 mmol) and triethylamine (TEA) (17 mL, 0.12 mmol) was dissolved in dry DCM (5 mL) in an eggplant-shaped bottle and placed on ice bath. When the temperature of the reaction system reached 0 °C, EDCI (22.95 mg, 0.12 mmol) was added, and then the ice bath was

removed. After reacting at r.t. for 24 h under protection of nitrogen, the reaction mixture was washed with 5% citric acid, distilled water and saturated brine successively. The organic phase was collected and dried over anhydrous Na_2SO_4 and purified by silica gel column chromatography (eluent: dichloromethane/methanol = 8/1 (*v*/*v*)). (b) Deprotection: the product was dissolved in hydrogen chloride/dioxane (4 mL) on ice bath. After stirring vigorously at r.t. for 30 min, the solvents were removed under reduced pressure to give 38.9 mg DoGo21. Yield: 23%. The synthetic route and characterizations are shown in Scheme S1.3 and Figure S1.5 and Figure S1.6, respectively.

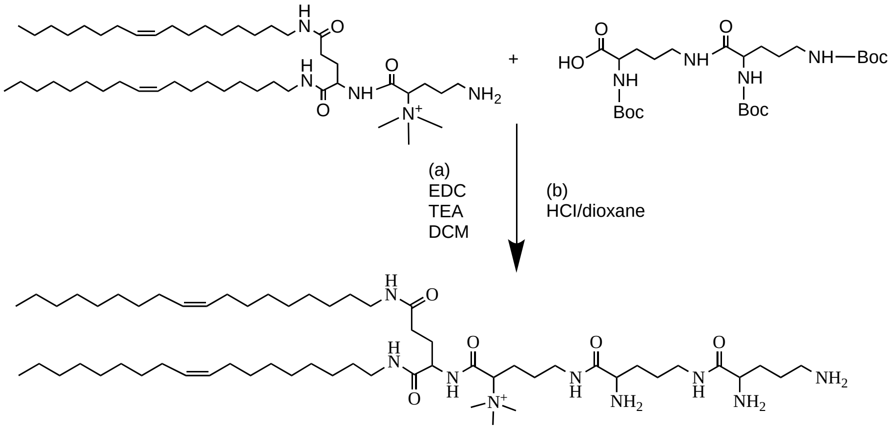

Scheme S1.3 Synthesis of DoGo21.

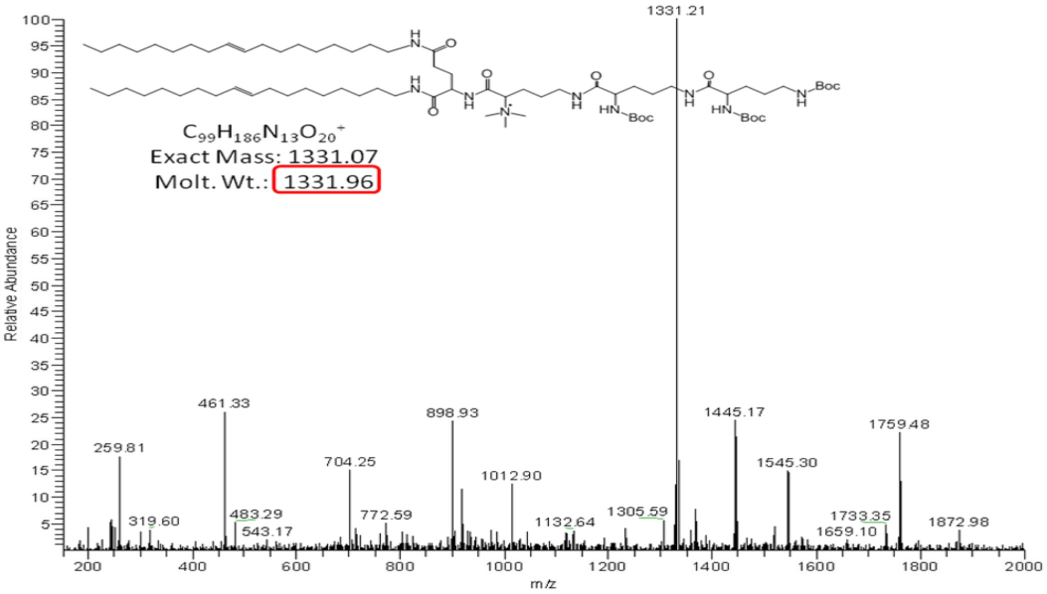

Figure S1.5 MS Spectrum of of DoGo21 (before deprotection).

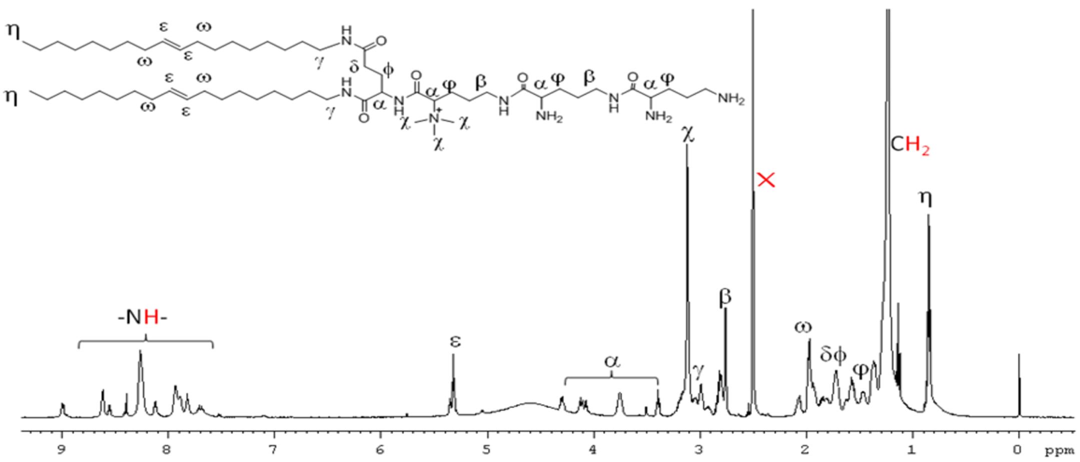

Figure S1.6 ^{1}HNMR Spectrum of DoGo21 (Solvent: DMSO-d6).

1.2. Synthesis of DoGo31

Synthesis of intermediate 1 of DoGo31:

(a) Cbz-Orn(Boc)-OH (100 mg, 0.27 mmol), PyBOP (170.4 mg, 0.33 mmol) and of HOBt (44.3 mg, 0.33 mmol) was dissolved in 5 mL of dry DMF in a 100 mL eggplant-shaped flask, and stirred on ice bath for 1 h, and then moved to r.t. condition. DoGo1 (212.0 mg, 0.33 mmol) and DIPEA (55 μL, 0.33 mmol) were added to the reaction mixture, followed by stirring at r.t. for 24 h under the protection of N_2. After the reaction, the DMF was evaporated by rotary evaporation, and separation and purification was carried out by silica gel column chromatography (eluent: DCM/methanol = 10/1 (*v*/*v*)). The separated product was rotary evaporated and dried under vacuum to give 238 mg intermediate 1. Yield: 88.7%. (b) Deprotection: 1/1 volume ratio of TFA (6 mL)/DCM (6 mL) was added to the intermediate 1, and the solution was vigorously stirred at r.t. for 0.5 h. After the reaction was completed, the solvents were removed by evaporation to give 239 mg deprotected intermediate 1. Yield: 100%. The synthetic route and characterizations are shown in Scheme S1.4 and Figure S1.7 and Figure S1.8, respectively.

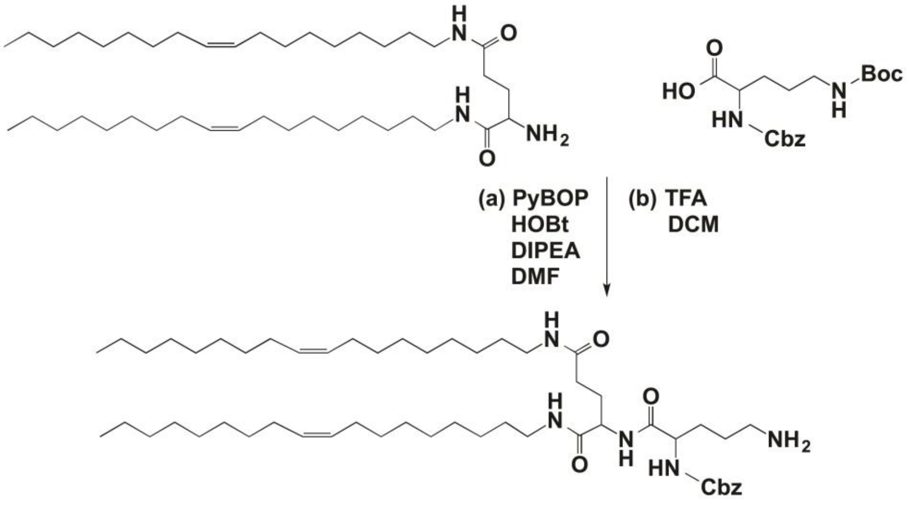

Scheme 1.4 Synthesis of intermediate 1 of DoGo31.

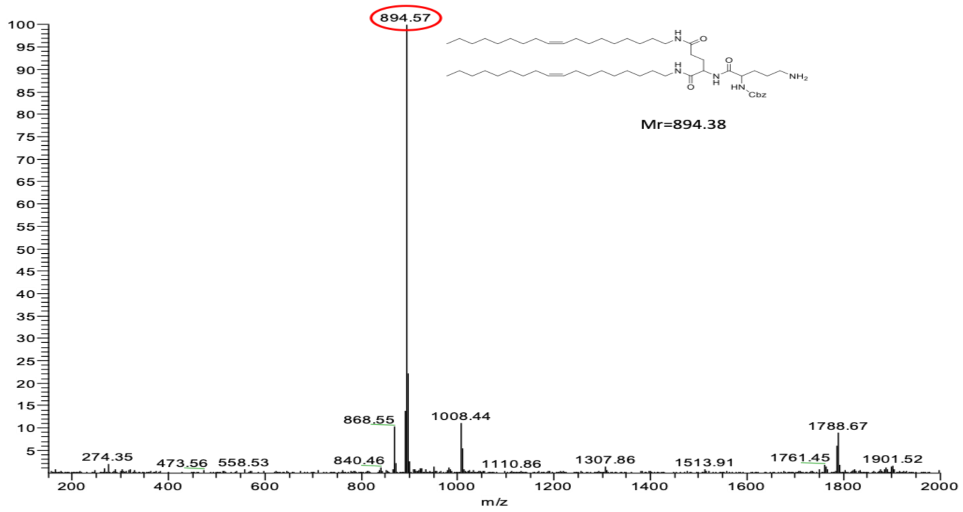

Figure S1.7 MS Spectrum of Intermediate 1 of DoGo31.

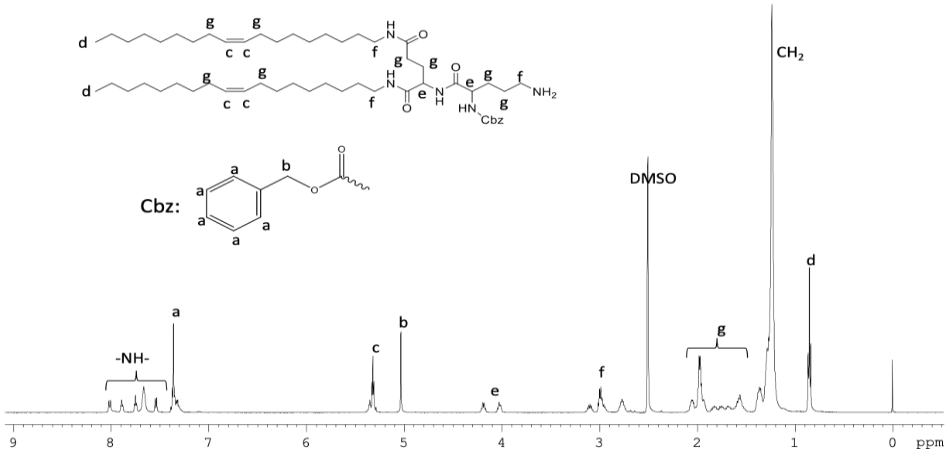

Figure S1.8 ^{1}HNMR Spectrum of Intermediate 1 of DoGo31 (Solvent: DMSO-d$_6$).

Synthesis of intermediate 2 of DoGo31:

(a) Cbz-Orn(Boc)-OH (88.0 mg, 0.24 mmol), PyBOP (151.0 mg, 0.29 mmol) and HOBt (39.0 mg, 0.29 mmol) was dissolved in 5 mL of dry DMF in a 100 mL of eggplant-shaped bottle, and reacted on ice bath for 1 h, and then moved to r.t. Intermediate 1 (217.0 mg, 0.24 mmol) and DIPEA (48 μL, 0.29 mmol) were dissolved in 3 mL of DMF and added into the reaction mixture, and then reacted at r.t. for 24 h under the protection of N_2. After the reaction, the DMF was evaporated, and the separation and purification were carried out by silica gel column chromatography (eluent: DCM/methanol = 8/1 (*v*/*v*)). The separated product was rotary evaporated and dried under vacuum to give 199 mg intermediate 2. Yield: 67%. (b) Deprotection: 1/1 ratio of TFA (6mL)/DCM (6 mL) was added to the intermediate 2, and the reaction mixture was vigorously stirred at r.t. for half an hour. After the solvents were removed, the product was dried under vacuum to give 201 mg deprotected intermediate product 2. Yield: 100%. The synthetic route and characterizations are shown in Scheme S1.5 and Figure S1.9 and Figure S1.10, respectively.

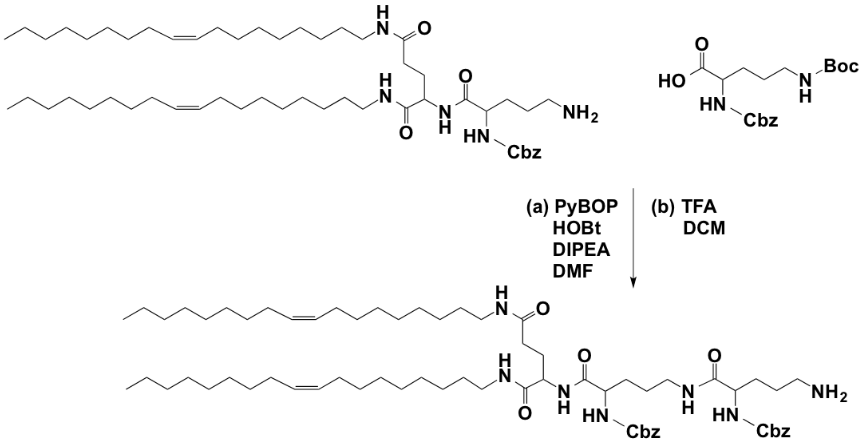

Scheme 1.5 Synthesis of intermediate product 2 of DoGo31.

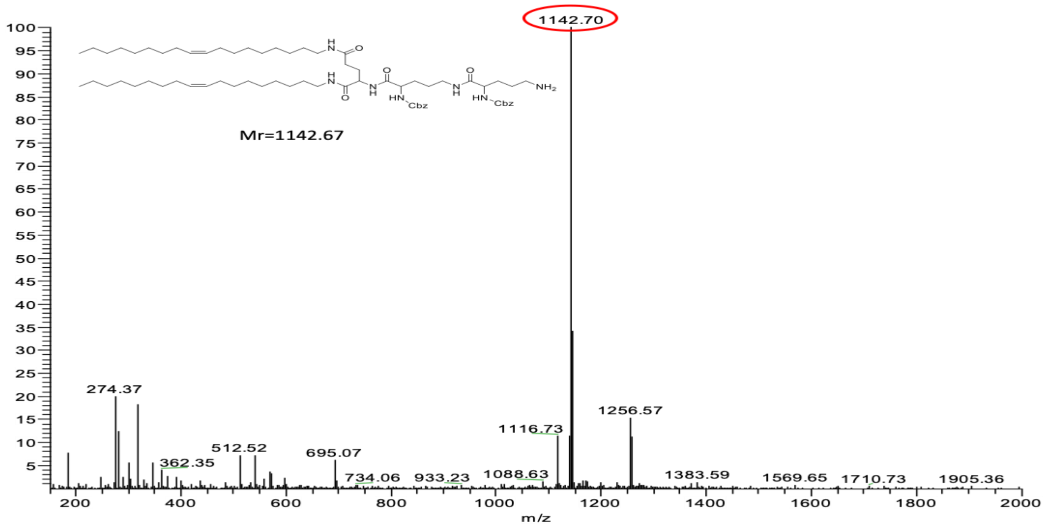

Figure S1.9 MS spectrum of Intermediate product 2 of DoGo31.

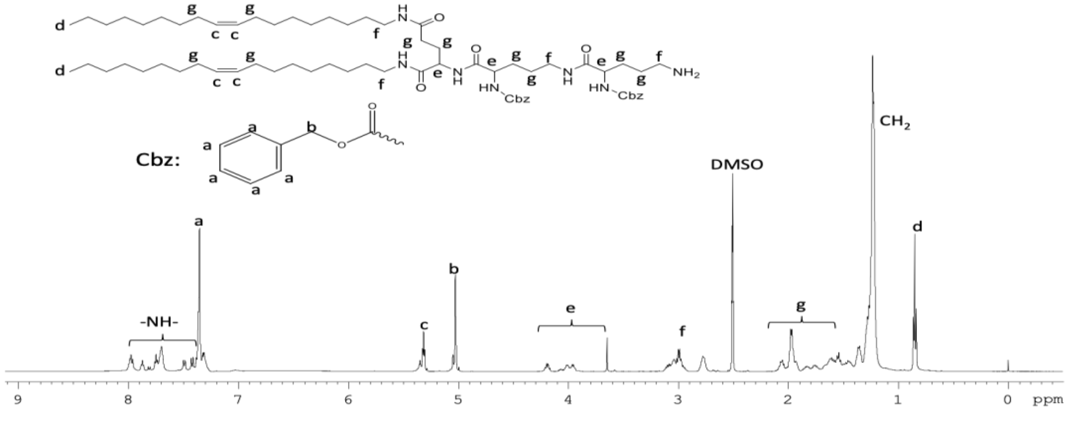

Figure S1.10 ^{1}HNMR spectrum of Intermediate product 2 of DoGo21 (Solvent: DMSO-d6).

Synthesis of intermediate 3 of DoGo31.

(a) Cbz-Orn(Boc)-OH (59.0 mg, 0.16 mmol), PyBOP (99.0 mg, 0.19 mmol) and HOBt (26.0 mg, 0.19 mmol) were dissloved in 4 mL of dry DMF in a 100 mL eggplant-shaped bottle, and reacted on ice bath for 1 h, and then placed at r.t. The intermediate 2 (184.0 mg, 0.16 mmol) and DIPEA (31.0 μL, 0.19 mmol) were dissolved in 5 mL of DMF, and added in to the reaction mixture, then stirred at r.t. for 24 h under the protection of N_2. After the reaction, the DMF was evaporated by rotary evaporation, and separation and purification was performed by silica gel column chromatography (eluent: DCM/methanol = 8/1 (*v*/*v*)).

The separated product was evaporated and dried under vacuum to give 129 mg intermediate 3. Yield: 54%. (b) Removal of Boc group: 1/1 ratio of TFA (6 mL)/DCM (6 mL) was added to intermediate 3, and the reaction was vigorously stirred at room temperature for 0.5 h. After the reaction was completed, the product was dried under vacuum to give 128 mg deprotected intermediate 3. Yield: 100%. The synthetic route and characterizations are shown in Scheme S1.6 and Figure S1.11 and Figure S1.12, respectively.

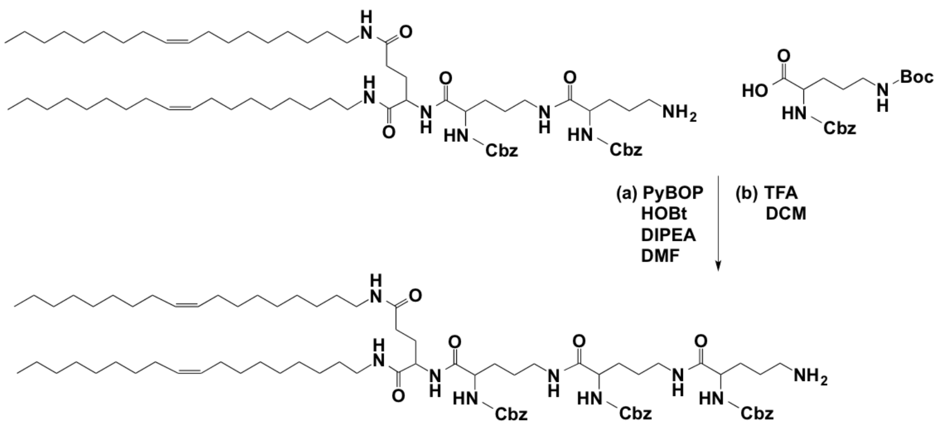

Scheme 1.6 Synthesis of intermediate 3 of DoGo31.

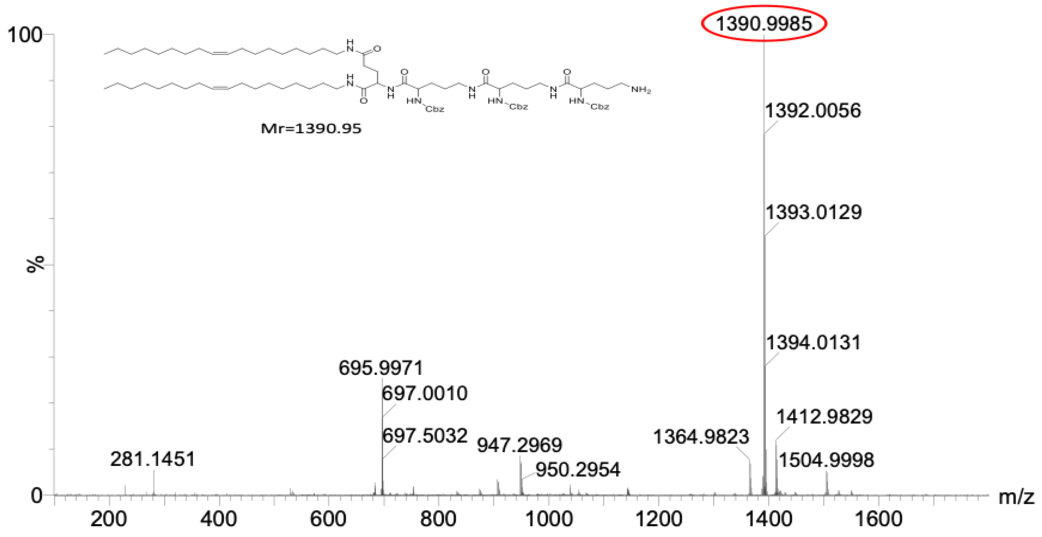

Figure S1.11 MS Spectrum of Intermediate 3 of DoGo21.

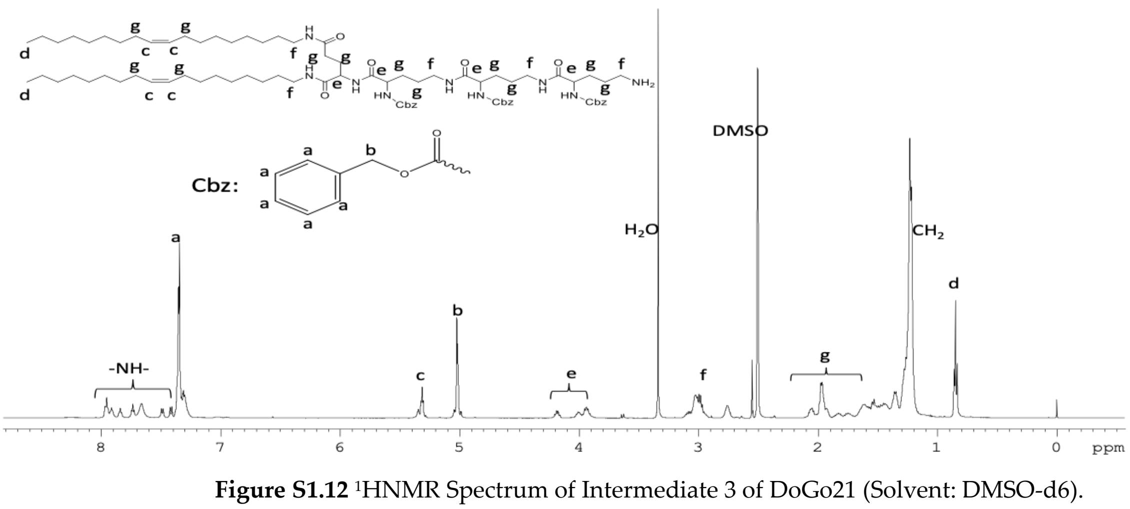

Figure S1.12 ^{1}HNMR Spectrum of Intermediate 3 of DoGo21 (Solvent: DMSO-d6).

Synthesis of DoGo31:

(a) Intermediate 3 (121 mg, 0.09 mmol) and K_2CO_3 (120.0 mg, 0.87 mmol) were stirred in 5 mL of CH_3I under a nitrogen atmosphere at 45 ^{0}C for 6 h. Then, CH_3I was removed, and the product was dissolved in DCM, followed by washing with water three times. The product was further separated and purified by silica gel column chromatography (eluent: DCM/methanol = 6/1 (*v*/*v*)). The separated product was collected by rotary evaporation and dried under vacuum to give 96 mg DoGo31. Yield: 75.4%. (b) Removal of Cbz group: DoGo31 was dissolved in 1:1 substance ratio of TFA:33%HBr/CH3COOH. The solution was stirred for 12 h. Finally, the product was dried under vacuum to give 94 mg DoGo31. Yield: 100%. The synthetic route and characterizations are shown in Scheme 1.7 and Figure S1.13 and Figure S1.14, respectively.

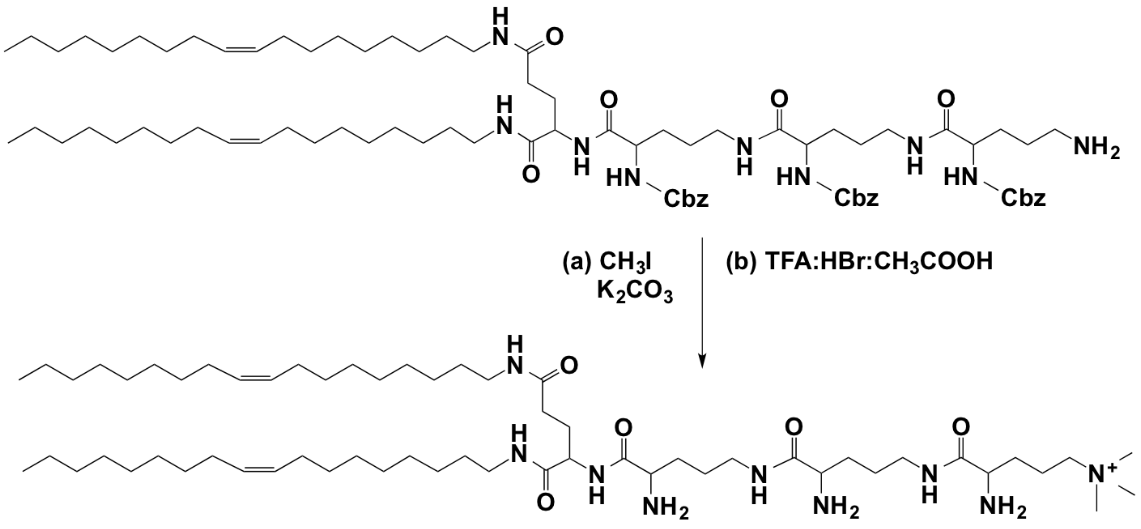

Scheme 1.7 Synthesis of DoGo31.

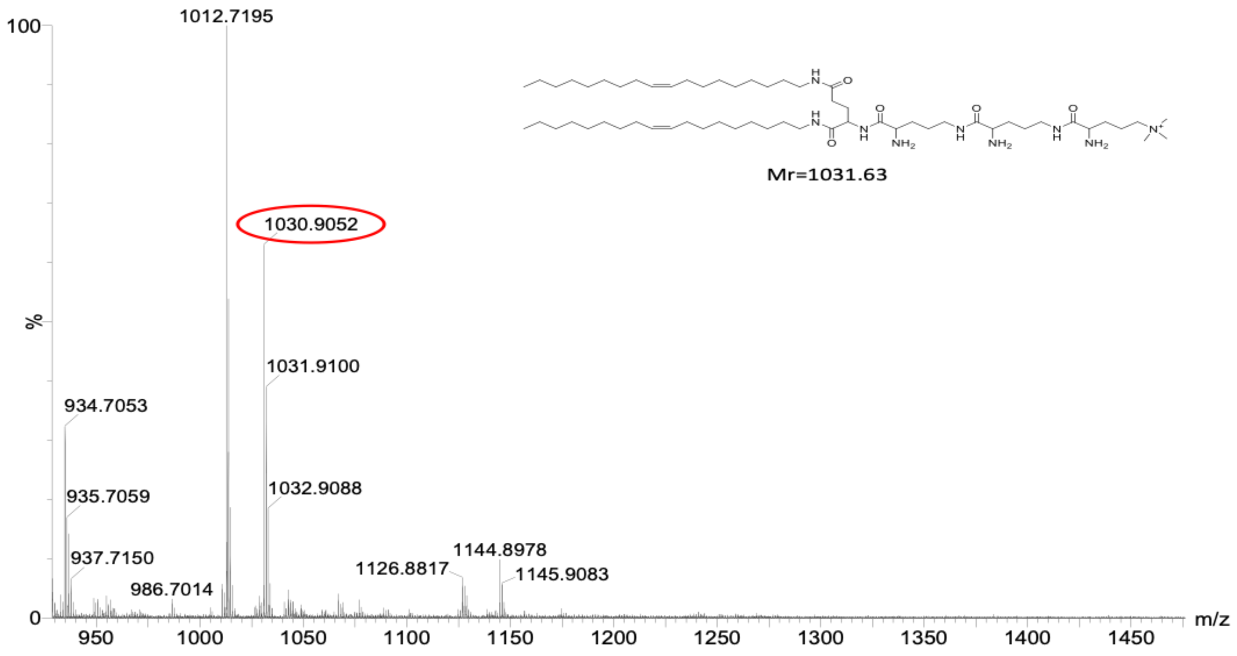

Figure S1.13 MS spectrum of DoGo31.

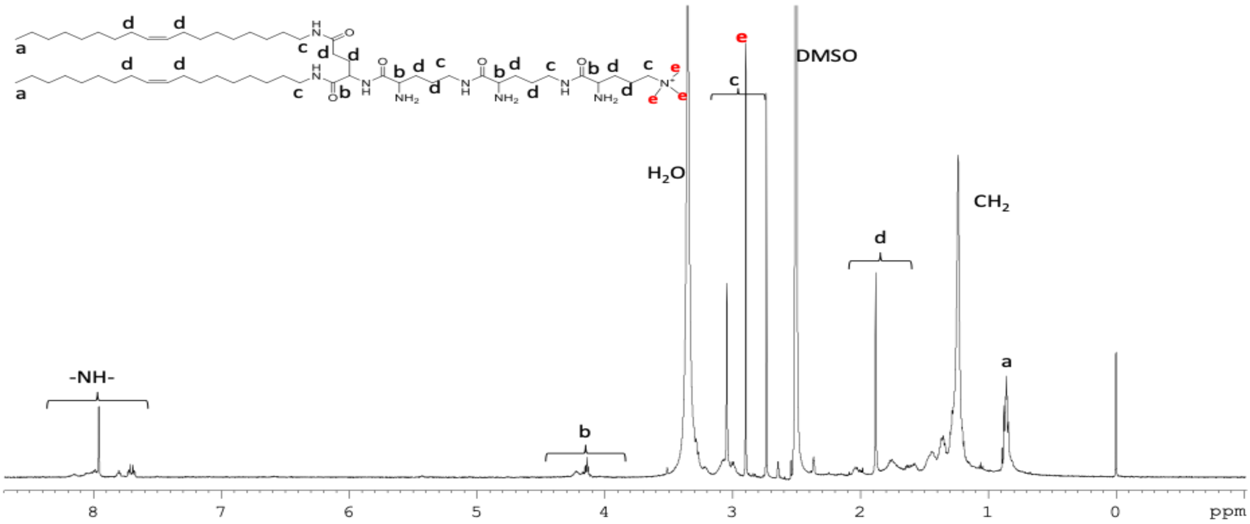

Figure S1.14 ^{1}HNMR Spectrum of DoGo31 (Solvent: DMSO-d6).

1.3. Synthesis of DoGo211

Synthesis of intermediate 1:

(a) Boc-Orn(Cbz)-OH (51.0 mg, 0.14 mmol), PyBOP (88.0 mg, 0.17 mmol) and HOBt (23.0 mg, 0.17 mmol) were dissolved in 3 mL of dry DMF in 100 mL of eggplant-shaped bottle, and reached for 1 h on ice bath. After adding intermediate 2 of DoGo31 (obtained from Scheme S1.5, 163.0 mg, 0.14 mmol) and DIPEA (28 μL, 0.17 mmol), the reaction was carried out at r.t. for 24 h under the protection of N_2. Then, DMF was evaporated, and purification was carried out by silica gel column chromatography (eluent: DCM/methanol = 8/1 (*v*/*v*)). The separated product was rotary evaporated and dried under vacuum to give 135 mg intermediate 1. Yield: 65%. (b) Removal of Boc group: 1/1 ratio of TFA (6 mL)/DCM (6 mL) was added to the intermediate 1, and the solution was vigorously stirred at room temperature for half an hour. After the reaction was completed, the final product was dried under vacuum to give 134 mg deprotected intermediate 1. Yield: 100%. The synthetic route and characterizations are shown in Scheme S1.8 and Figure S1.15 and Figure S1.16, respectively.

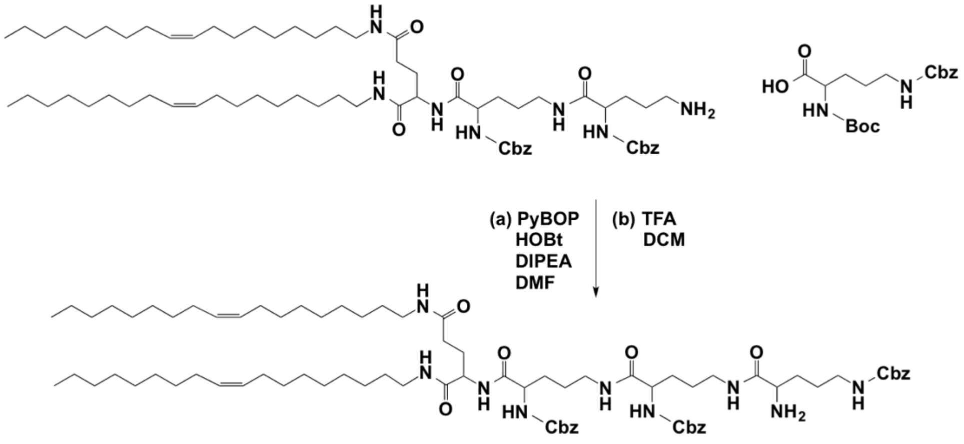

Scheme 1.8 Synthesis of intermediate 1 of DoGo211.

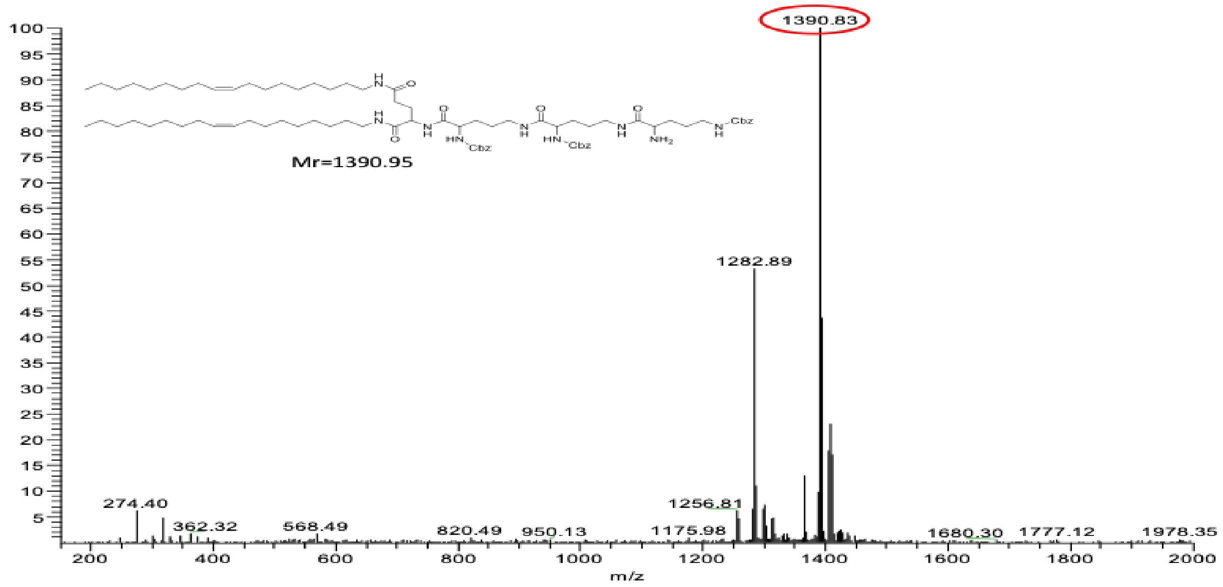

Figure S1.15 MS spectrum of Intermediate 1 of DoGo211.

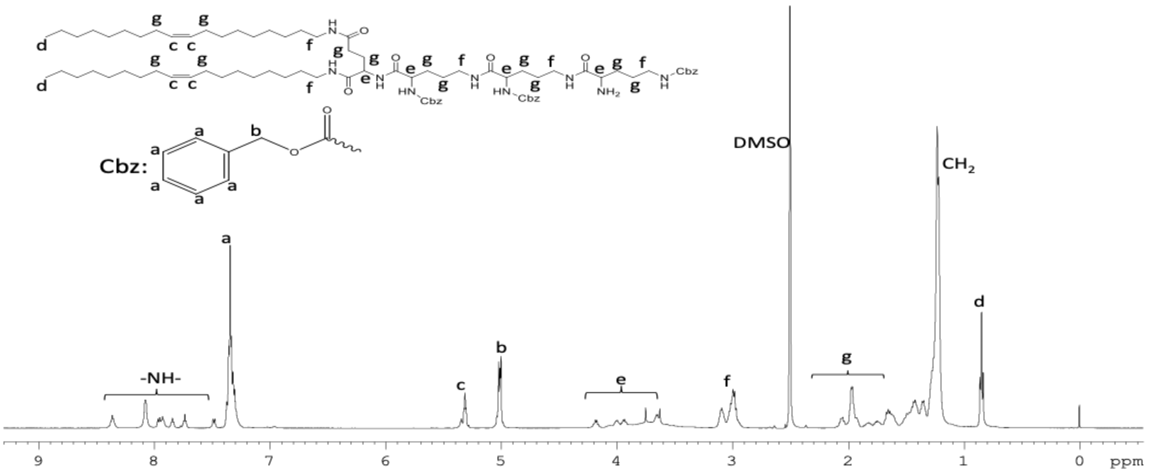

Figure S1.16 ^{1}HNMR Spectrum of Intermediate 1 of DoGo211 (Solvent: DMSO-d6).

Synthesis of DoGo21:

(a) Intermediate 1(126 mg, 0.091 mmol), K_2CO_3 (126 mg, 0.91 mmol) and 5 mL of CH_3I were srirred under nitrogen atmosphere at 45 °C for 6 h. Then, CH_3I was removed and the resulting syrup was dissolved in DCM and washed with water three times. Next, the product was separated and purified by silica gel column chromatography (eluent: DCM/methanol = 6/1 (*v*/*v*)). The separated product was concentrated by rotary evaporation and dried under vacuum to give 74 mg DoGo21. Yield: 57%. (b) Removal of Cbz group: DoGo21 was dissolved in 1:1 substance ratio of TFA:33%HBr/CH_3COOH. Then, the solution were stirred for 12 h. Finally, the product was dried under vacuum to give 70 mg deprotected DoGo21. Yield: 100%. The synthetic route and characterizations are shown in Scheme S1.9 and Figure S1.17 and Figure S1.18, respectively.

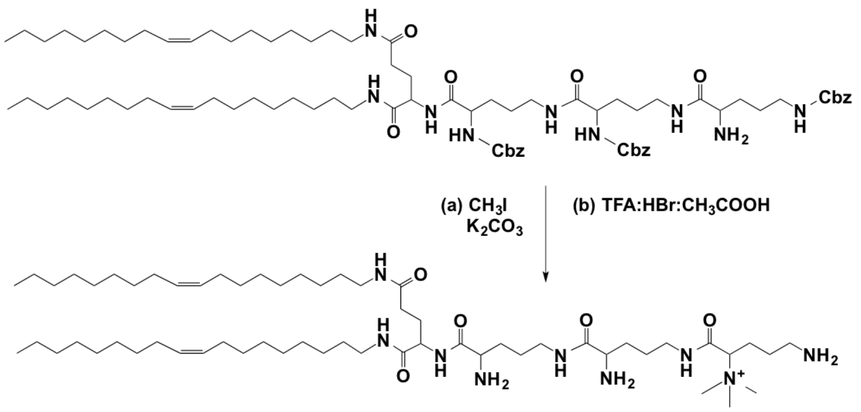

Scheme 1.9 Synthesis of DoGo21.

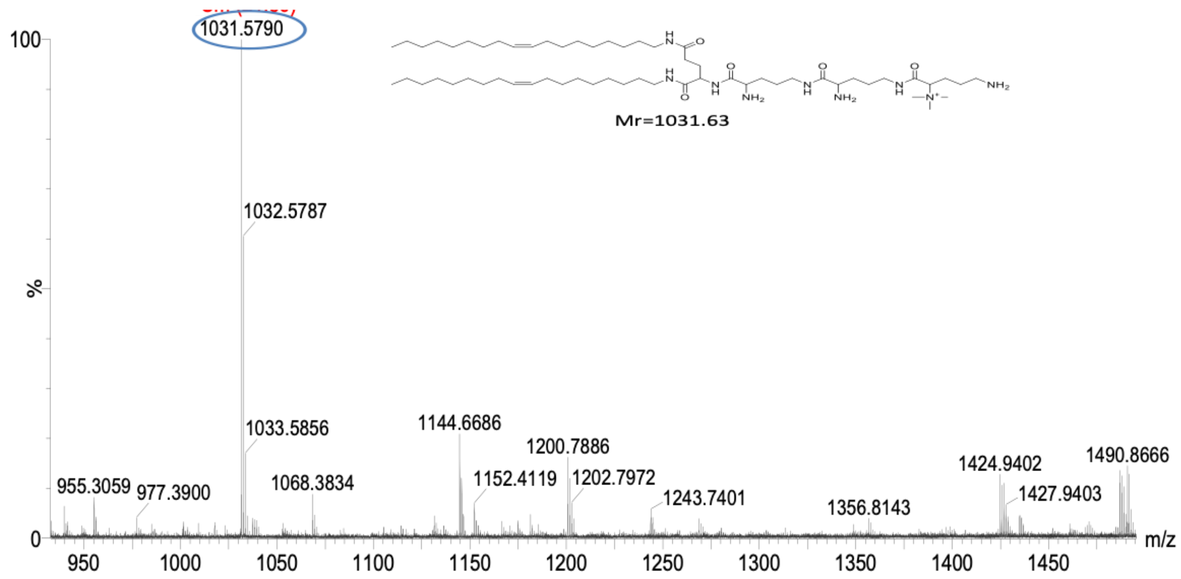

Figure S1.17 MS spectrum of DoGo21.

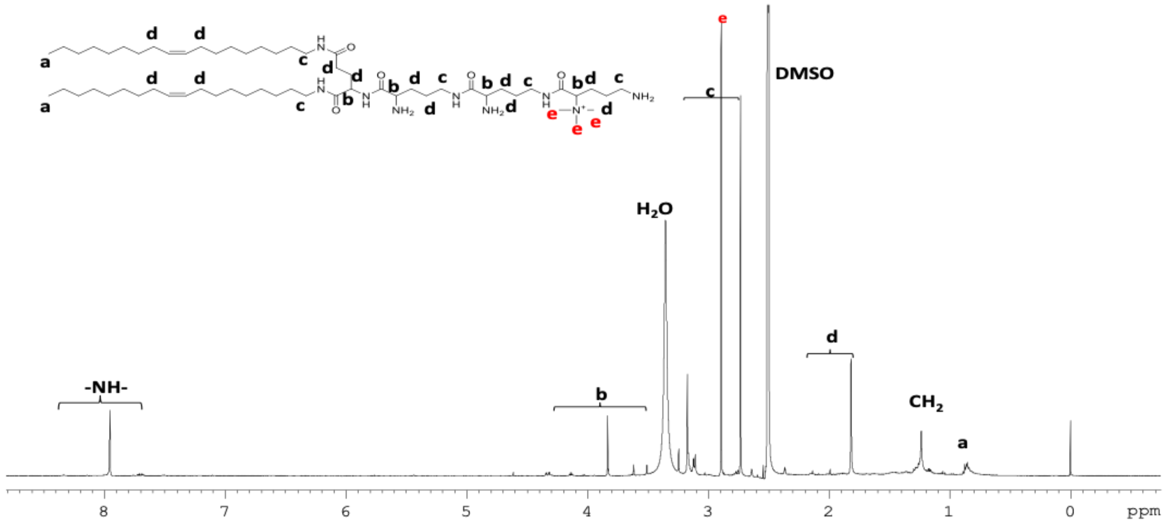

Figure S1.18 ^{1}HNMR spectrum of DoGo21 (Solvent: DMSO-d$_6$).

1.4. Synthesis of DoGo310

(a) Boc-Orn[(Boc-Orn(Boc-Orn(Boc))]-OH (100 mg, 0.13 mmol) in DMF (1 mL), DoGo1 (90 mg, 0.13 mmol) in DCM (3 mL), and DIPEA (27.5 uL, 0.16 mmol) were mixed in a round bottom flask and cooled on an ice bath. Then PyBOP (102 mg, 0.19 mmol) was

added and, after the ice bath was removed, the reaction mixture was stirred for 24 h under nitrogen protection. Then the solvent was removed under reduced pressure and the resulting syrup was resuspended in DCM, followed by washing with 5% citric acid solution for three times. The product was concentrated and purified by silica gel chromatography using DCM/MeOH (15:1) as eluents to give 147 mg unprotected DoGo310. Yield: 80.6%.

(b) Deprotection: DoGo310 was stirred in DCM/TFA to give final product DoGo310. The synthetic route and characterizations are shown in Scheme S1.10 and Figure S1.19 and Figure S1.20, respectively.

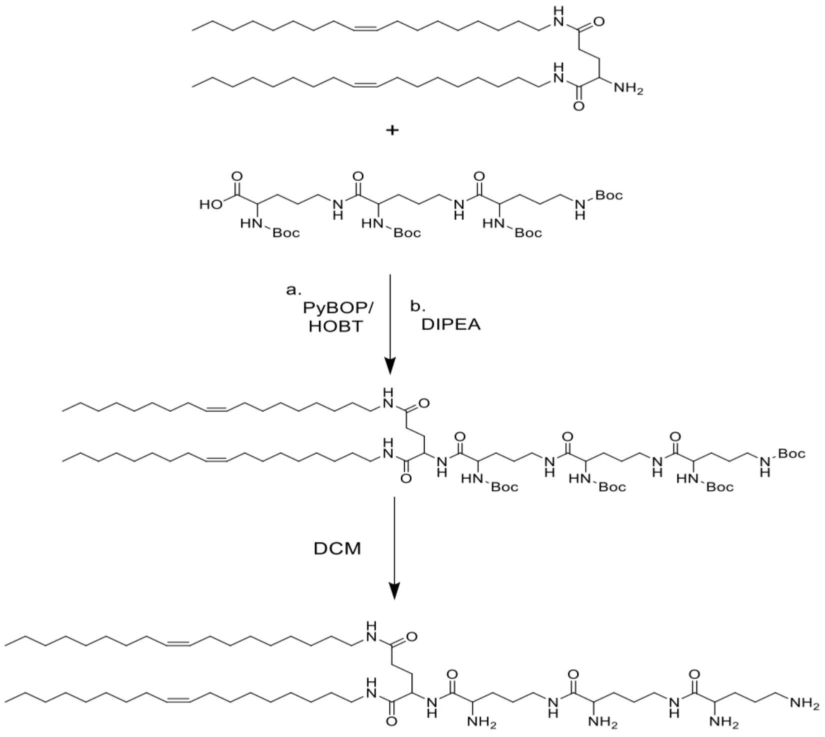

Scheme 1.10 Synthesis of DoGo310.

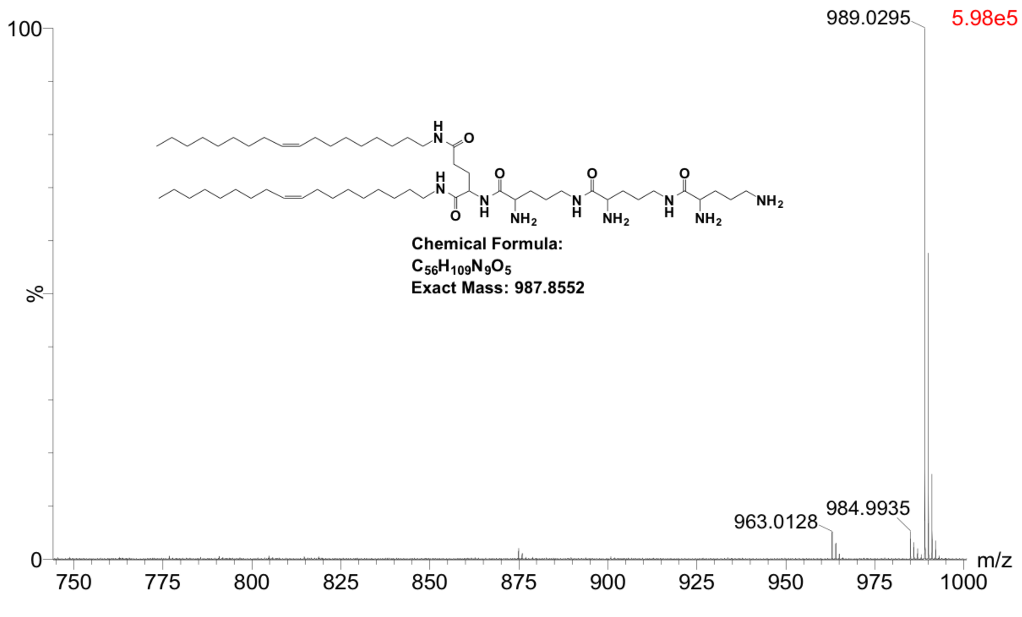

Figure S1.19 MS spectrum of DoGo310.

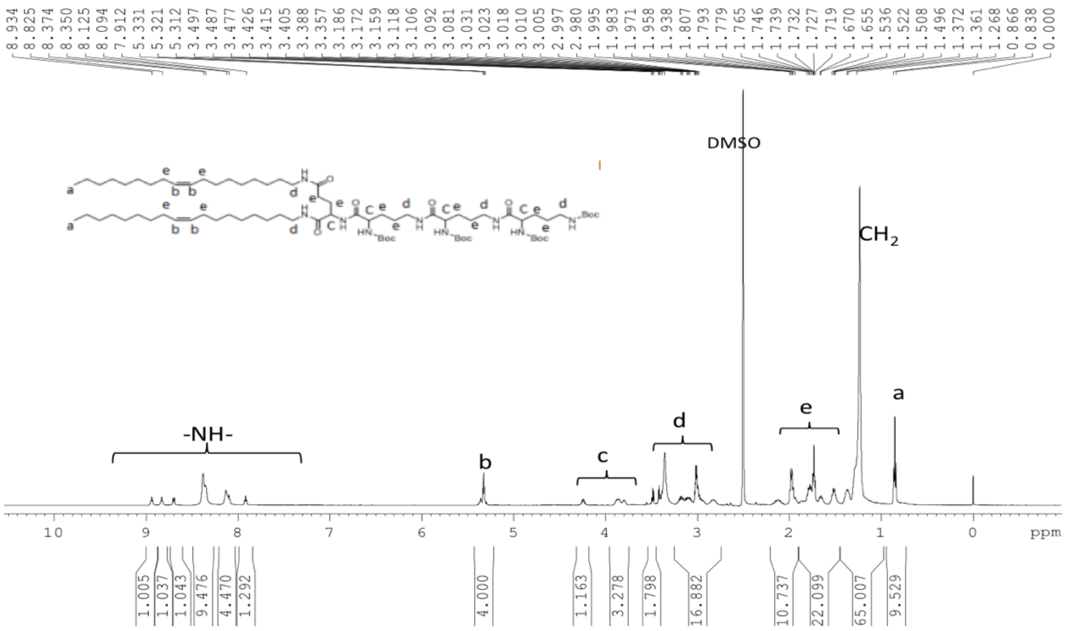

Figure S1.20 ^{1}HNMR spectrum of DoGo310 (Solvent: DMSO-d6).

2. Supporting Information Table

Table S1. The sequences of the primers used for RT-qPCR analysis.

Gene	Direction	Sequence
Mouse TLR4	forward	5′- ggactctgatcatggcactg-3′
	reverse	5′-ctgatccatgcattggtaggt-3′
mouse β-actin	forward	5-ctaaggccaaccgtgaaaag-3′
	reverse	5′-accagaggcatacagggaca-3′
mouse TNFα	forward	5′-tcttctcattcctgcttgtgg-3′
	reverse	5′-ggtctgggccatagaactga-3′
mouse IL-1β	forward	5′-agttgacggaccccaaaag-3′
	reverse	5′-agctggatgctctcatcagg-3′
mouse IL-10	forward	5′-cagagccacatgctcctaga-3′
	reverse	5′- tgtccagctggtcctttgtt-3′
mouse IL-4	forward	5′-catcggcattttgaacgag-3′
	reverse	5′-cgagctcactctctgtggtg-3′
mouse Arg1	forward	5′-gaatctgcatgggcaacc-3′
	reverse	5′-gaatcctggtacatctgggaac-3′
mouse CD206	forward	5′-tcattggaagatccactctgg-3′
	reverse	5′-cagcgcttgtgatcttcattatag-3′
mouse TGF-1β	forward	5′-ctgggcaccatccatgac-3′
	reverse	5′-cagttcttctctgtggagctga-3′
mouse iNOS	forward	5′-ctttgccacggacgagac-3′
	reverse	5′-tcattgtactctgagggctgac-3′
mouse ROS	forward	5′-ctctggagcaaaccactctgt-3′
	reverse	5′-ggctgttgttgtaccaatcca-3′
IP-10	forward	5′-gctgccgtcattttctgc-3′
	reverse	5′- tctcactggcccgtcatc-3′

3. Supporting Information Figures

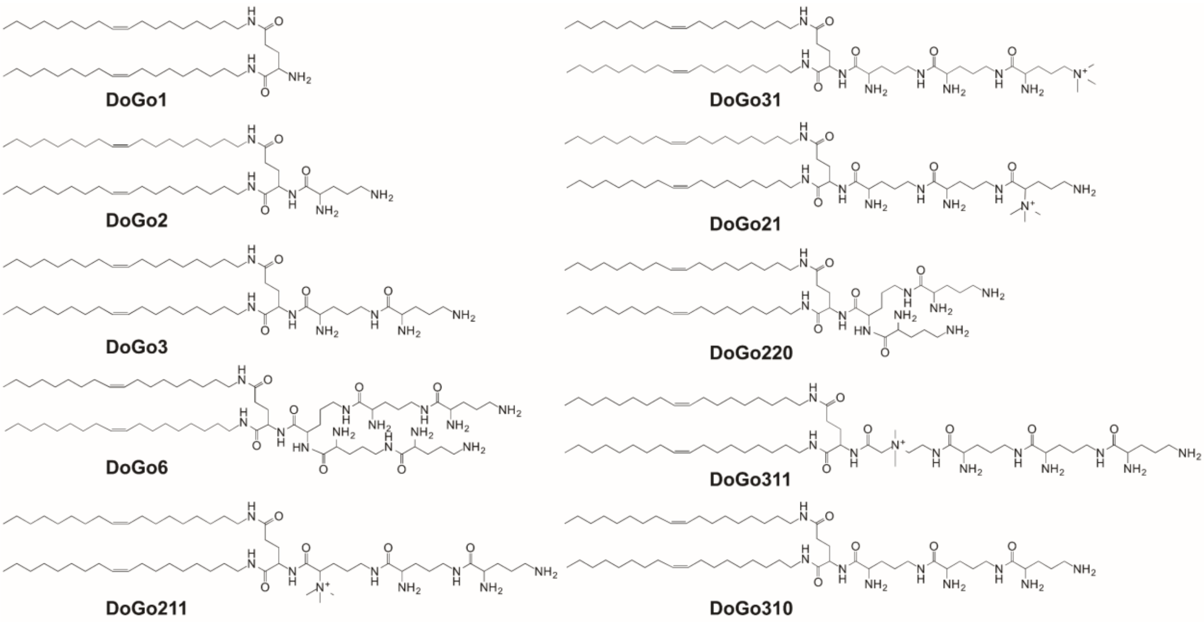

Figure S2. Chemical structure of the designed DoGo peptidomimetics.

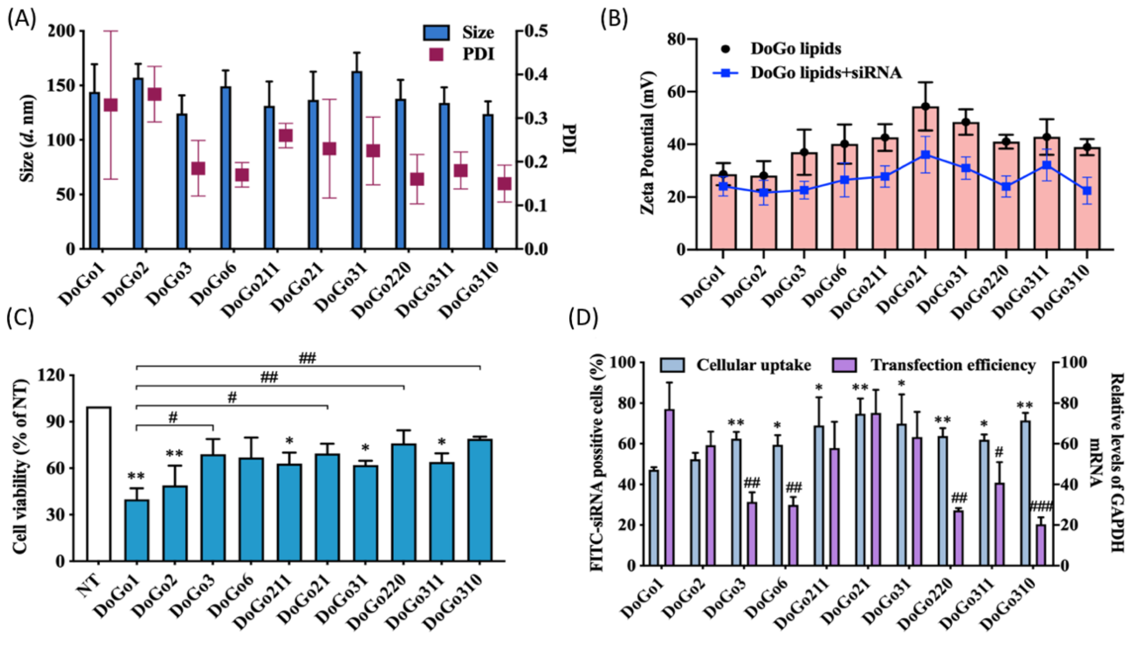

Figure S3. Initial screening of DoGo lipids for cytotoxicity and transfection efficiency. (**A**) Particle size of DoGo lipids. DoGo lipids were suspended in PBS buffer (pH 7.4), respectively, and the particle size was measured by dynamic light scattering; (**B**) The zeta potential of DoGo lipids before and after complexing with siRNA; (**C**) Cytotoxicity of DoGo lipids. DoGo lipids bearing more than 3 amino groups on their head group showed less toxicity to BV2 cells compared with the ones that have one or two amino groups; NT, nontreated group. (**D**) siRNA delivery efficiency of DoGo lipids. BV2 cells were transfected with FITC-labeled siRNA (for cellular uptake assay, left Y axis) or GAPDH siRNA (for RNAi efficiency, right Y axis) complexed to individual DoGo lipids. DoGo220 and DoGo310 showed 80–85% inhibiting of the targeted gene expression at a dose of 10 nM of siRNA (concentration of DoGos: 20 μg/mL). All data are presented as mean ± SD from triplicate experiments; $^{*,\#}p < 0.05$, $^{**,\#\#}p < 0.001$, $^{***,\#\#\#}p < 0.0005$ compared to untreated cells.

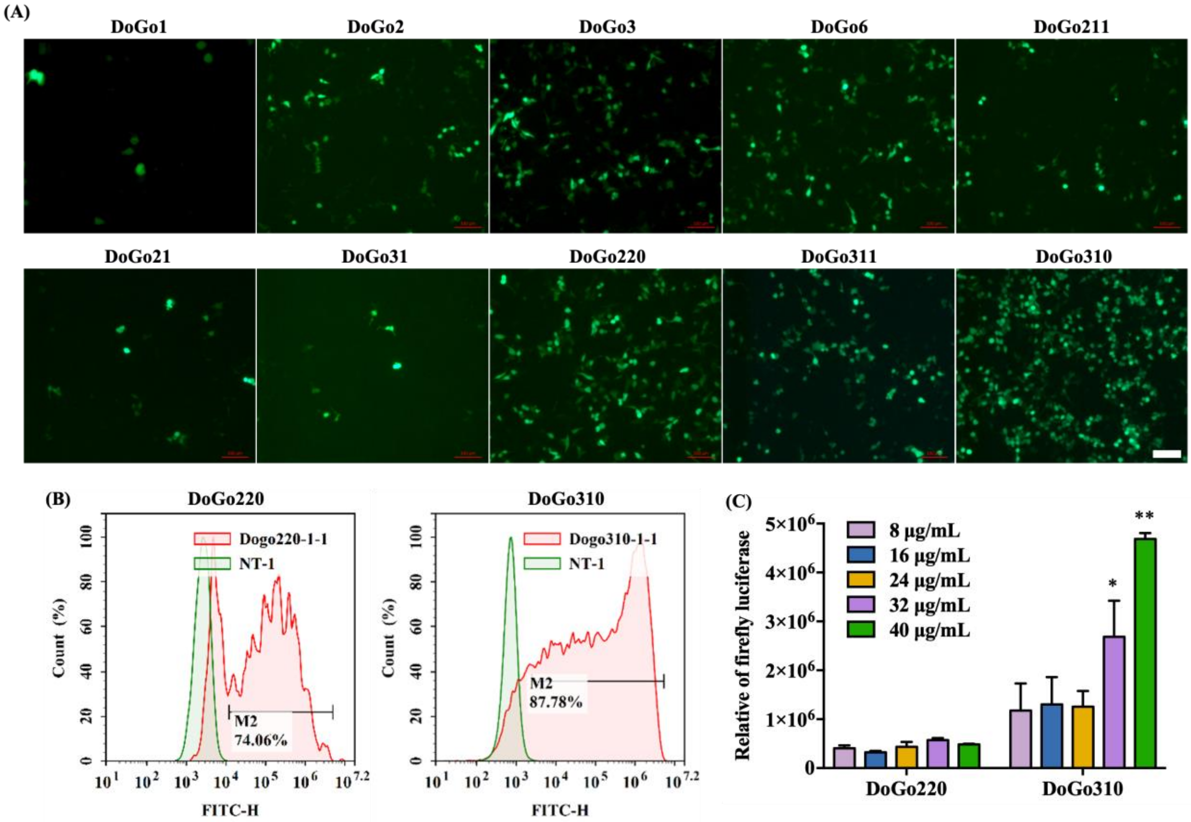

Figure S4. Evaluation of plasmid transfection efficiency of the peptidomimetics. (**A**) 239T cells were treated with pcDNA-eGFP plasmid complexed to DoGo peptidomimetics (structures are shown in Figure S2). 24 h after transfection, the fluorescence signal of the cells was observed under fluorescence microscope. (**B**) Quantification of transfection efficiency by flow cytometry. Plasmid transfection efficiency of the 2 peptidomimetics (DoGo220 and DoGo310) that showed the strongest fluorescence signals in the cells after transfected with complex of GFP-labelled plasmid DNA compared to others. (**C**) The transfection efficiency of DoGo220 and DoGo310 at an increasing concentration was further compared by transfecting 293T cells with pGL3-SV40 plasmid complexed to DoGo220 and DoGo310, respectively. The result showed that the transfection efficiency of DoGo310 is significantly higher than that of DoGo220.

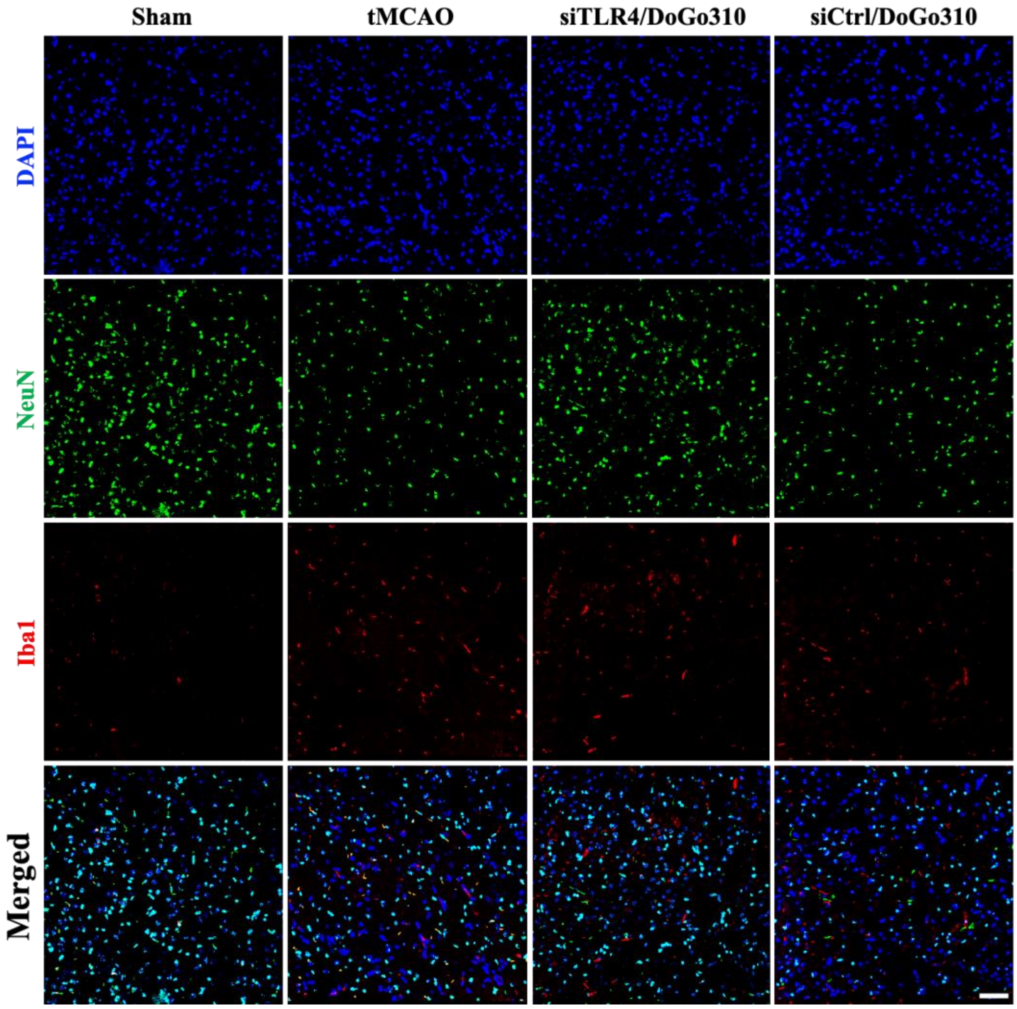

Figure S5. siTLR4/DoGo310 LNP mediated knockdown of TLR4 rescued NeuN⁺ cells (neurons) in peri-infarct region of tMCAO model mouse. Immunohistochemistry analysis of peri-infarct region from sham, siCtrl/DoGo310 and siTLR4/DoGo310 LNP treated mouse model of tMCAO. Red, Iba-1⁺ cells; Green, NeuN⁺ cells. Scale bar = 100 μm.
